# Segmental BIA-derived body composition in children and adolescents aged 5–18 years: age- and sex-specific reference values and limitations in obesity

**DOI:** 10.1038/s41430-026-01788-1

**Published:** 2026-08-01

**Authors:** Klara Böker, Mandy Vogel, Annelie Grundmann, Wieland Kiess

**Affiliations:** 1 Leipzig University, Medical Faculty, University Hospital for Children and Adolescents Leipzig, Center for Pediatric Research (CPL), LIFE Child, Leipzig, Germany; 2 German Center for Child and Adolescent Health (DZKJ), Partner Site Leipzig/Dresden, Leipzig, Germany

**Keywords:** Epidemiology, Techniques and instrumentation, Education

## Abstract

**Background/Objectives:**

Bioimpedance analysis (BIA) is a non-invasive method for estimating fat-free mass (FFM) and fat mass (FM) via resistance (Rz) and reactance (Xc). Despite its potential, its usefulness and accuracy in children remain controversial.

**Subjects/Methods:**

We obtained body composition measurements from 2954 visits involving 1547 participants (aged 5–21 years) using segmental BIA measurements and skinfold-based equations (Slaughter et al.). Four independent analyses were conducted: (1) agreement of Rz and Xc measurements between body segments was assessed; (2) sex- and age-specific reference curves for height-normalized resistance (Rz/H) and reactance (Xc/H) were developed; (3) associations between changes in BMI-SDS, Rz/H, and Xc/H (SDS) were examined; and (4) FM estimates derived from the proprietary algorithm, an alternative formula-based BIA algorithm, and skinfold-based equations were compared with age adjustment.

**Results:**

Segmental Rz/H and Xc/H measurements showed “excellent” (>0.9) intraclass correlations for all segments. Concordance correlation coefficients indicated variable agreement between methods. Age- and sex-specific Rz/H and Xc/H percentile curves showed similar progressions across body segments. Rz/H (SDS) and Xc/H (SDS) showed a clear association with BMI-SDS, with lower values in participants with obesity. Estimated FFM and FM showed discrepancies between estimation methods depending on weight status rather than age.

**Conclusion:**

Rz/H and Xc/H showed consistent age-related patterns but were strongly influenced by BMI-SDS. While agreement between FM estimates was good in normal-weight children, substantial discrepancies were observed in those with obesity. These findings suggest that current BIA-based estimation approaches may be unreliable in children and adolescents with obesity.

## Introduction

With over 400 million (20%) affected in 2022, childhood overweight and obesity are a worldwide public health issue [1]. As childhood obesity develops in early childhood [2] and likely persists into adulthood [3], implying severe comorbidities, it is a serious issue for affected individuals and for the public good. As BMI cannot accurately measure excess fat mass, precise obesity diagnostics are needed, especially in childhood [4, 5].

Bioimpedance analysis (BIA) is an affordable, non-invasive, and safe method for evaluating body composition [6]. It can be used as a screening tool or at the bedside. In adolescents and adults, BIA-based estimations of fat and fat-free mass correlate strongly with dual-energy X-ray absorptiometry (DXA) [7]. However, its use in pediatrics is controversial. BIA is currently not recommended for children under 24 months of age [8], and its accuracy is questionable because prediction equations are tissue-, population-, and device-specific [7, 9], leading to disparate formulas often based on a few hundred participants.

To translate segmental BIA into a clinically viable tool for pediatric populations, four interdependent questions must be addressed: [1] the reliability of segmental measurements to ensure consistent data acquisition; [2] the development of age- and sex-specific reference standards for interpreting raw impedance values; [3] the validation of these values against longitudinal changes in adiposity indicators; and [4] the contextualization of BIA performance relative to alternative methods. This study integrates these dimensions into a unified framework, demonstrating how raw BIA parameters—when anchored to developmental norms and validated against clinical outcomes—can mitigate current limitations in pediatric body composition assessment. By addressing these questions collectively, we provide a comprehensive foundation for the practical implementation of BIA in diverse clinical and research settings.

Accordingly, this study evaluated the reliability and agreement of segmental resistance (Rz) and reactance (Xc) measurements obtained with a segmental single-frequency BIA (SF-BIA) device, established age- and sex-specific percentile curves for height-normalized BIA parameters (Rz/H and Xc/H), examined their associations with BMI-SDS, and compared different approaches for estimating body composition, including two BIA calculation algorithms and a skinfold-based estimation approach. Agreement between fat mass estimates was additionally assessed using age-stratified analyses.

## Subjects and methods

### Study population

The data were collected as part of the LIFE Child Study, which focuses on the development of lifestyle diseases in a large pediatric cohort [10, 11]. There were 1,615 subjects (804 girls, 811 boys) aged 4–21 years with at least one bioimpedance measurement and triceps/subscapular skinfold measurements. We excluded duplicate measurements (*n* = 6) and those from children <5 years of age (*n* = 19) due to their limited representation. Furthermore, we excluded all subjects suffering from diseases influencing body composition, especially water balance (heart, kidney, liver, and cancer diseases, *n* = 87), subjects taking respective medications (diuretics, antidiuretics, thyroid hormones, antiepileptic drugs, or antidepressants, *n* = 218), and subjects with implausibly low BMI-SDS (BMI-SDS < –4: *n* = 2). Implausible bioimpedance measurements were excluded (phase angle >10° (*n* = 5), phase angle difference left/right >1.5° (*n* = 9)). The final sample included 2954 measurements from 1547 subjects aged between 5 and 21 years (Supplementary Fig. 1).

### Measurement methods

Measurements of height, weight, and skinfolds were conducted following standardized procedures. Detailed information can be found in the Supplement, section Anthropometric Measures. Pubertal development was assessed according to Tanner [12, 13]. Tanner stage (TS) 1 was considered prepubertal, TS 2–4 pubertal, and TS 5 postpubertal. BIA measurements were conducted between 2012 and 2022 using the “BIACORPUS RX 4000” (MEDI CAL HealthCare GmbH, Karlsruhe, Germany) and the accompanying proprietary software “BodyComp Professional”. This phase-sensitive SF-BIA device measured Rz and Xc segmentally for the trunk and all four limbs at a frequency of 50 kHz. To minimize variability, standardized measurement conditions were implemented: To ensure that bodily fluids were distributed evenly, individuals had to lie horizontally for at least five minutes before the measurement, as recommended [14]. No mandatory fasting period was imposed [15, 16] and participants were instructed to avoid vigorous physical activity prior to measurement. Standardization included ensuring no contact between the thighs, torso, and arms during measurement to avoid current shunting, and controlled ambient room temperature to limit impedance variability. If necessary, the extremities were fanned out using pillows or sheets. The inner electrodes were attached between the bony prominences in the center of the wrists. The outer electrodes were positioned near the fingertips, at least 3 cm away from the center electrode. To ensure the minimum distance required, the outer electrode was adhered to the palm of the hand if the needed distance could not otherwise be achieved. The electrodes were placed in a similar manner on the feet (Supplementary Fig. 2). The four electrode positions resulted in measurements for six segments: RARF, RALA, RFLF, RALF, and LARF, LALF, where “R”/“L” denote right/left and “A”/“F” arm/foot. We hypothesized the symmetrical axes RARF/LALF and RARF/LARF as comparable measurement segments, allowing us to use segmental analyses to evaluate measurement consistency across body segments and thereby validate the reliability of segmental BIA data.

Therefore, in this study, a total of three sets of body composition estimates were derived. First, measurements were taken using the BIACORPUS RX 4000, which outputs Rz and Xc. FFM and FM were then calculated using the device’s proprietary formula. Second, Rz and Xc outputs from the BIACORPUS RX 4000 were used to calculate FM and FFM according to Gätjens et al.‘s calculation algorithm [17]. This comparison was included to evaluate the consistency between two algorithms that were developed for large German cohorts and based on phase-sensitive SF-BIA (50 kHz) devices.

Finally, FM was estimated using skinfold thickness measurements according to Slaughter et al.‘s method [18], to compare BIA-derived estimates with an alternative, widely used field method for assessing body composition in pediatric populations. The calculation formulas used by Slaughter et al. and Gätjens et al. are listed in the Supplement.

### Statistical analysis

Using German reference data BMI was computed and converted to BMI Standard Deviation Scores (BMI-SDS) [19]. Weight classes were defined as: extreme underweight: BMI-SDS < –1.88, underweight: –1.88 < BMI-SDS < –1.28, normal weight: –1.28 < BMI-SDS < 1.28, overweight: 1.28 < BMI-SDS < 1.88, obese: 1.88 < BMI-SDS < 2.58, and severely obese: BMI-SDS ≥2.58 [19].

Bland-Altman analyses and the overall concordance correlation coefficient (OCCC), concordance correlation coefficient (CCC), and intraclass correlation coefficient (ICC) were applied to estimate agreement in Rz/Xc between body halves and body diagonals [20–23]. OCCCs and CCCs were classified as: ≥0.9 (“excellent”), <0.9 and ≥0.7 (“good”), < 0.7 and ≥0.5 (“moderate”), and <0.5 (“low”) [24]. The CCC was used for pairwise comparisons and the OCCC for comparisons between multiple observers, when comparing the different segmental BIA derivatives. ICC interpretation followed Koo and Li [25].

After standardizing Rz and Xc for body height in cm (H) [15], we estimated age- and sex-specific references from 2435 BIA measurements (1255 children and adolescents: 624 girls, 631 boys) using generalized additive models for location, shape, and scale [26], excluding subjects with underweight or obesity. Linear regression models were used to estimate associations between BMI-SDS changes and changes in resistance/reactance-SDS between consecutive visits (follow-up time 10–24 months), excluding subjects with ∆BMI-SDS/year >1.5.

Rz and Xc were incorporated into the formulas proposed by Gätjens et al., along with height, weight, and age, to facilitate the comparison of FFM/FM between the two BIA algorithms [17]. FM according to Slaughter et al. was calculated considering age, sex, and TS [18]. We compared the different FM estimates (BIACORPUS, Gätjens, and Slaughter) using Bland-Altman statistics [20] and additionally performed age-stratified analyses to evaluate potential age-related biases.

The significance level was set to α = 0.05. 95% confidence intervals are reported where applicable. Data were analyzed using R (version 4.4.2) [27]. Visual representations were created using the ggplot2 or BodyMapR [28, 29]. The analysis code is available upon reasonable request from the corresponding author.

## Results

Table 1 shows participant characteristics. Inclusion and exclusion are visualized in Supplementary Fig. 1.

**Table 1:** Characteristics of our cohort stratified by sex, including body composition measurements.

	[ALL] *N* = 2954	female *N* = 1419	male *N* = 1535	p.overall
**Mean age, years**	12.5 (3.30)	12.6 (3.37)	12.4 (3.24)	0.063
**Sexual maturity rating (SMR) in Tanner stages, numeric**	2.83 (1.63)	3.29 (1.60)	2.27 (1.49)	<0.001
**Sexual maturity rating (SMR) in Tanner stages**				<0.001
T I: prepubescent	818 (33.9%)	308 (23.1%)	510 (47.1%)	
T II: early puberty	369 (15.3%)	162 (12.2%)	207 (19.1%)	
T III: midpuberty	273 (11.3%)	185 (13.9%)	88 (8.13%)	
T IV: advanced puberty	316 (13.1%)	192 (14.4%)	124 (11.4%)	
T V: postpuberty	639 (26.5%)	485 (36.4%)	154 (14.2%)	
**Mean height, cm**	155 (17.8)	154 (16.0)	157 (19.1)	<0.001
**Mean BMI, kg/m**^**2**^	20.8 (5.53)	21.1 (5.84)	20.6 (5.23)	0.013
**Mean BMI-SDS**	0.43 (1.30)	0.47 (1.34)	0.39 (1.26)	0.115
**Weight groups defined by BMI-SDS**				0.006
Extreme underweight	67 (2.27%)	30 (2.11%)	37 (2.41%)	
Underweight	144 (4.87%)	71 (5.00%)	73 (4.76%)	
Normal weight	1978 (67.0%)	950 (66.9%)	1028 (67.0%)	
Overweight	246 (8.33%)	111 (7.82%)	135 (8.79%)	
Obese	333 (11.3%)	144 (10.1%)	189 (12.3%)	
Severely obese	186 (6.30%)	113 (7.96%)	73 (4.76%)	
**Fat-free mass according to BIA, kg**	39.8 (13.3)	37.2 (10.6)	42.1 (14.9)	<0.001
**Fat mass according to BIA, kg**	12.4 (10.6)	14.4 (11.6)	10.7 (9.19)	<0.001
**Fat mass according to BIA, %**	21.3 (10.9)	24.7 (10.9)	18.2 (9.98)	<0.001
**Total body water according to BIA, l**	28.0 (9.56)	26.0 (7.30)	29.9 (10.9)	<0.001
**Mean skin fold thickness of the subscapular fold, mm**	12.3 (9.10)	12.6 (8.71)	12.0 (9.42)	0.061
**Mean skin fold thickness of the triceps fold, mm**	15.1 (7.87)	16.5 (7.40)	13.9 (8.09)	<0.001
**Fat mass according to Slaughter, %**	23.8 (12.0)	23.9 (9.38)	23.6 (14.5)	0.547

### Reliability of segmental BIA measurements

In general, both Rz/H and Xc/H reached “good” OCCCs (OCCC_Rz/H_ = 0.899, CI (0.893, 0.907); OCCC_Xc/H_ = 0.898, CI (0.891, 0.905)). However, for Rz/H, the horizontal derivatives RFLF and RALA showed lower CCC values. The OCCC_Rz/H_ increased to 0.986 (CI (0.985, 0.987)) when RFLF and RALA were omitted, and OCCC_Xc/H_ increased to 0.92 (CI (0.915, 0.926)). “Excellent” CCCs were reached between arm-to-leg derivatives representing the body halves (RARF/LALF: CCC_Rz/H_ = 0.96, CI (0.959, 0.964); CCC_Xc/H_ = 0.99, CI (0.988, 0.989)). Interestingly, for Xc, the CCC between the LARF derivative and the other arm-to-leg derivatives was considerably weaker (Supplementary Fig. 3). The general ICC also showed “excellent” agreement (ICC3 All-Rz/H: 0.97, CI (0.97, 0.97); All-Xc/H: 0.94, CI (0.94, 0.94)). Looking at the RARF/LALF and RARF/LARF segments also showed “excellent” agreement (ICC3 RARF/LALF Rz/H: 0.99, CI (0.99, 0.99); Xc/H: 0.96; CI (0.96, 0.97); ICC3 RALF/LARF: Rz/H: 0.98, CI (0.98, 0.99); Xc/H: 0.93, CI (0.93, 0.94)).

**Fig. 1:**
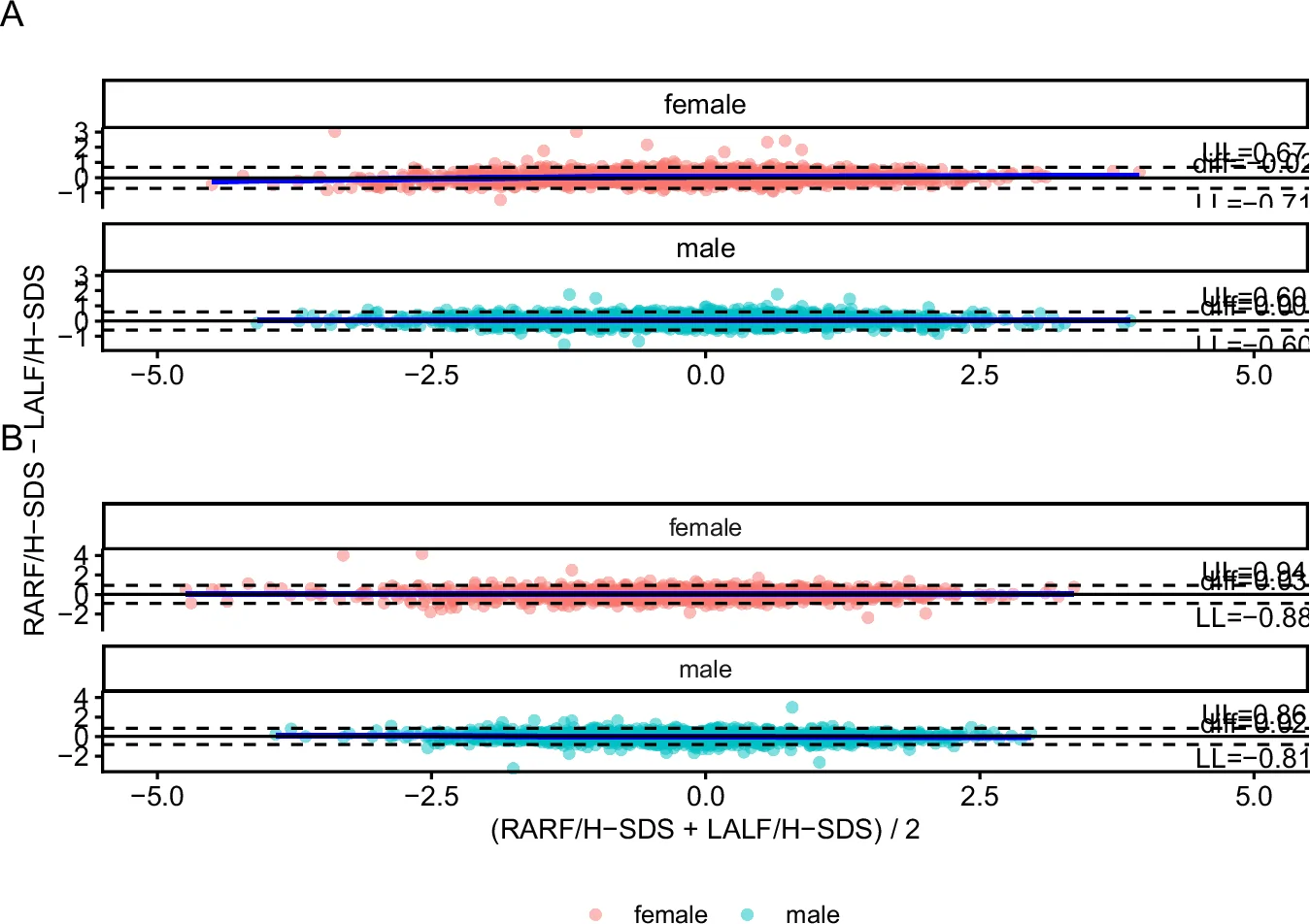
Results of Bland-Altman analyses comparing the Rz/H-SDS and Xc/H-SDS for the derivatives RARF and LALF. **A** No significant systematic differences were found for Rz/H (bias girls: –0.018, bias boys: 0.002). **B** Slight systemic bias in the Xc/H-SDS could be found for both sexes (bias girls: 0.027, bias boys: 0.024).

Bland-Altman analyses of Rz/H revealed that there were no systematic or proportionate biases between RALF/LARF or RARF/LALF. However, there was a slight systematic bias in the Xc/H-SDS for the RARF/LALF segments in both sexes. Systematic biases also existed for Xc/H-SDS values in girls. See Fig. 1, Supplementary Fig. 4, and Supplementary Table 13.

### Age- and sex-specific reference curves for Rz/H and Xc/H

**Fig. 2:**
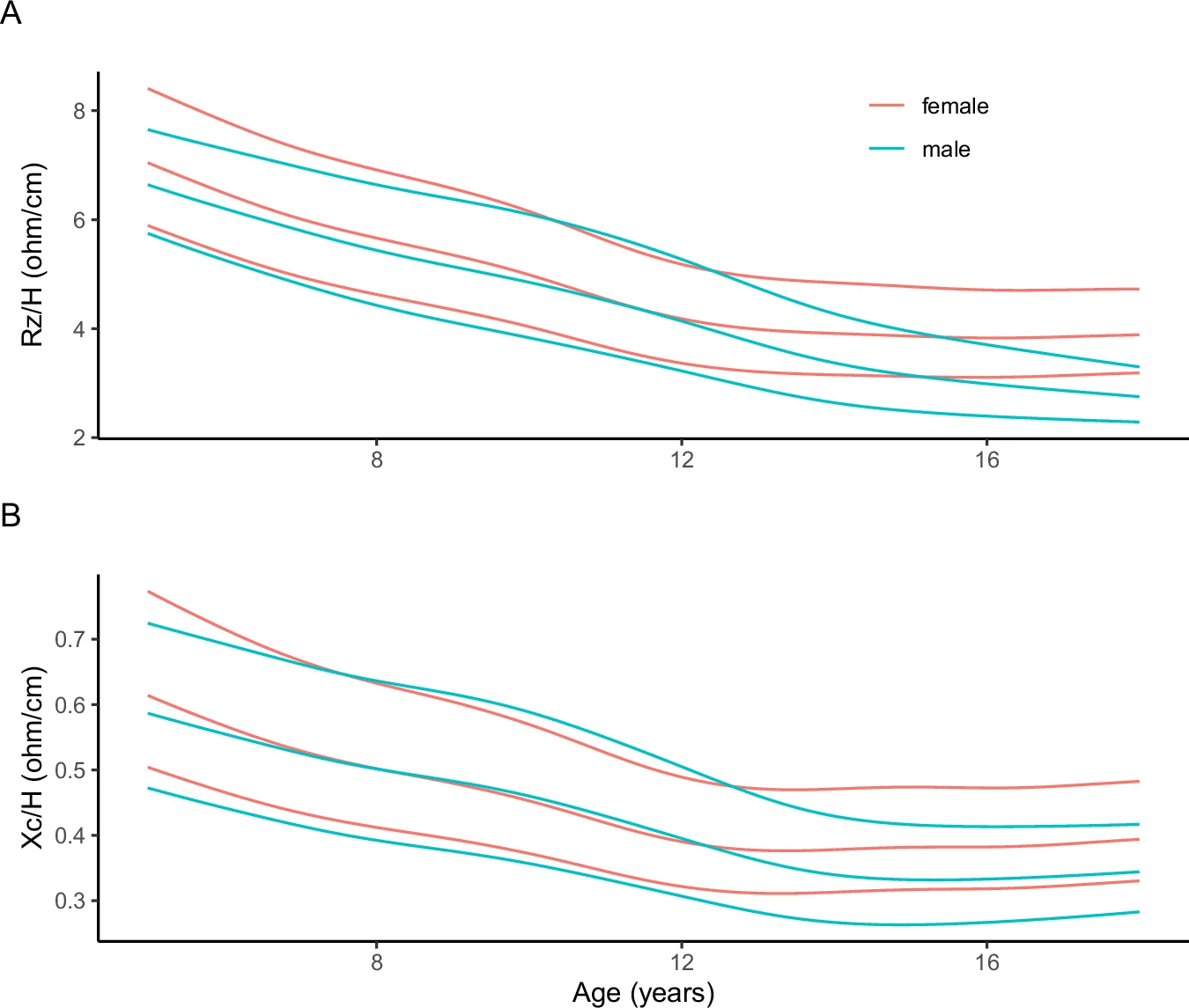
The 2.5th, 50th, and 97.5th percentile curves for Rz/H and Xc/H shown for the derivative RARF. The percentile curves for **A** Rz/H and **B** Xc/H showed similar patterns for boys and girls. Generally, resistance (Rz/H) and reactance (Xc/H) were similar in boys and girls until the age of 12. After that, girls showed higher values than boys.

Age progressions were similar across the six measurement segments. Figure 2 displays the percentile curves for Rz/H and Xc/H for RARF. Both metrics showed a decrease from age 5 across all percentiles. In boys, the decrease became less steep from age 15 but continued until the end of the observation period. In girls, the decline ended in a plateau at age 12 for both measures. In general, Rz and Xc were similar in boys and girls until age 12, and were then higher in girls (Supplementary Tables 1–12).

### Clinical associations with BMI-SDS

**Fig. 3:**
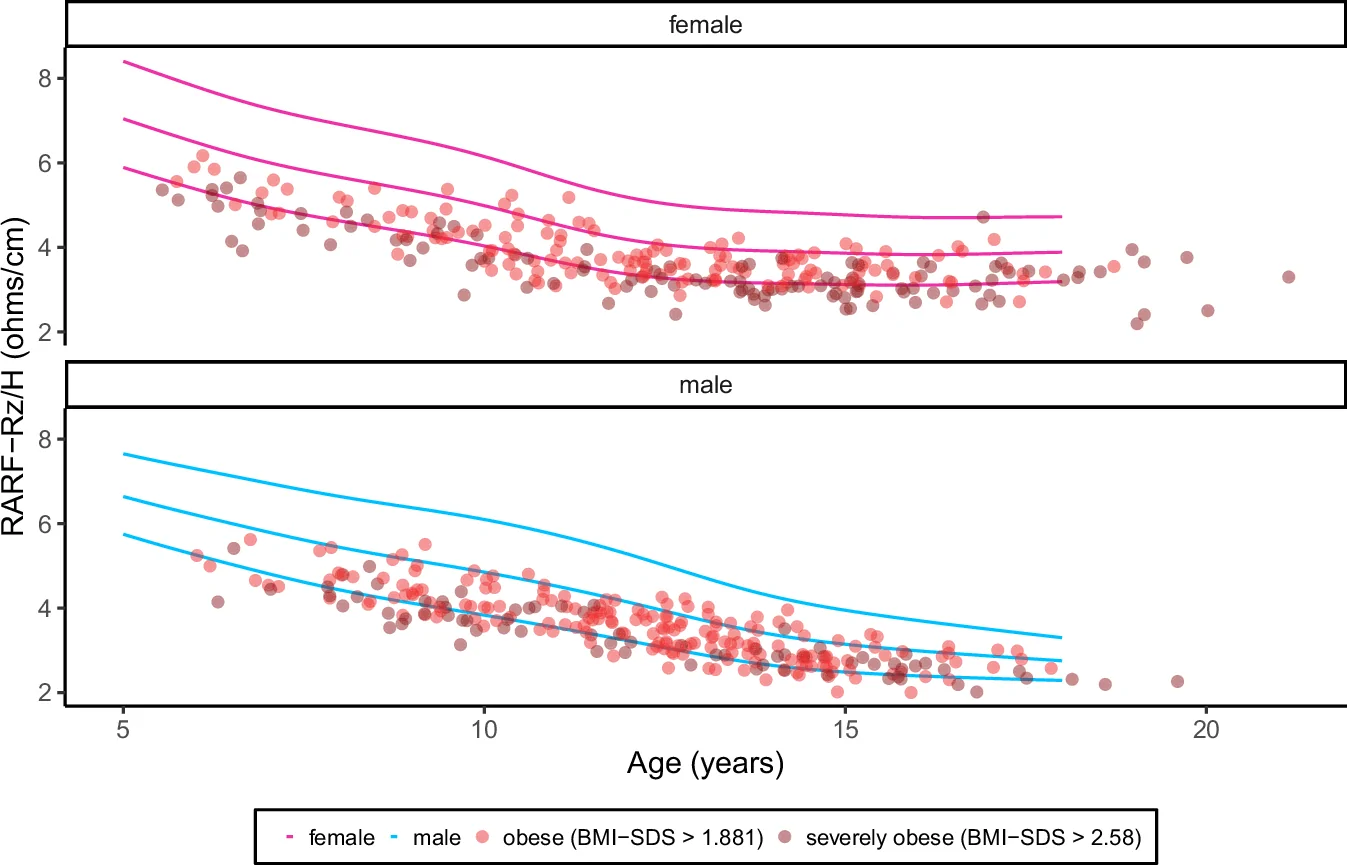
Comparison of Rz/H values in children and adolescents with obesity against reference values shown for the derivative RARF. Compared with the reference ranges derived from a non-obese study population, Rz/H values were lower for children and adolescents with obesity or severe obesity, with most values below the 50th percentile. The other derivatives showed similar patterns.

**Fig. 4:**
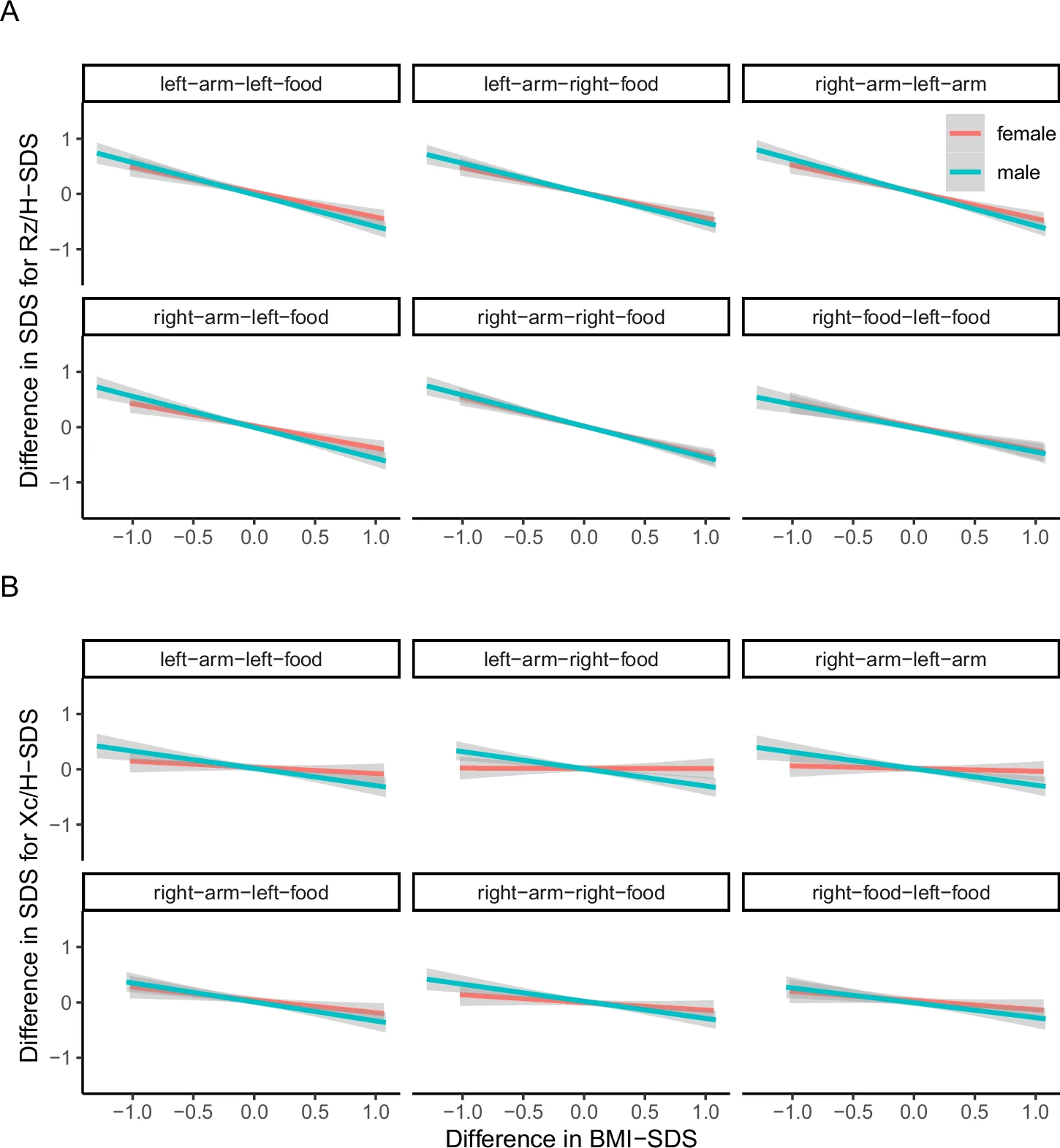
Associations between changes in Rz/H-SDS and Xc/H-SDS and changes in BMI-SDS. **A** We found strong inverse associations between changes in Rz/H-SDS and changes in BMI-SDS, even after adjusting for BMI-SDS at baseline. **B** Similar associations were seen to a lesser extent for Xc/H.

Overall, Rz/H and Xc/H values were lower in participants with obesity and severe obesity compared with the normal weight reference. Most values were below the corresponding age- and sex-specific medians, especially for Rz/H (Fig. 3 and Supplementary Fig. 5). Changes in Rz/H-SDS were strongly inversely associated with changes in BMI-SDS (adjusted for BMI-SDS at t0) with effect sizes between ß = –0.5 and ß = –0.6 (all *p* < 0.001, corresponding CI ranged from –0.7 to –0.4). Consistently, higher BMI-SDS was associated with lower Rz/H (see Fig. 4). Effects for Xc/H-SDS were inverse as well but considerably weaker, with effect sizes between ß = –0.2 SDS and ß = –0.3 (all ps <0.001, corresponding CI ranged from −0.43 to−0.10). For RFLF and LARF no significant association with Xc/H-SDS was found.

### Cross-method comparison of different estimation methods for body composition

We found substantial differences in FFM (kg, %), and FM (kg, %), between estimates for BIACORPUS RX 4000 and Gätjens et al.’s formulas [17]. FFM (kg and %) showed significantly higher values for the BIACORPUS algorithm for both sexes (Supplementary Table 13). Agreement depended on FM estimates (Supplementary Fig. 6). The stratified Bland-Altman plot (Supplementary Fig. 7) showed different biases for different FFM ranges as well.

Consistently, significantly lower FM (kg and %) estimates were found for BIACORPUS compared with Gätjens et al. (Supplementary Table 13, Supplementary Fig. 8).

**Fig. 5:**
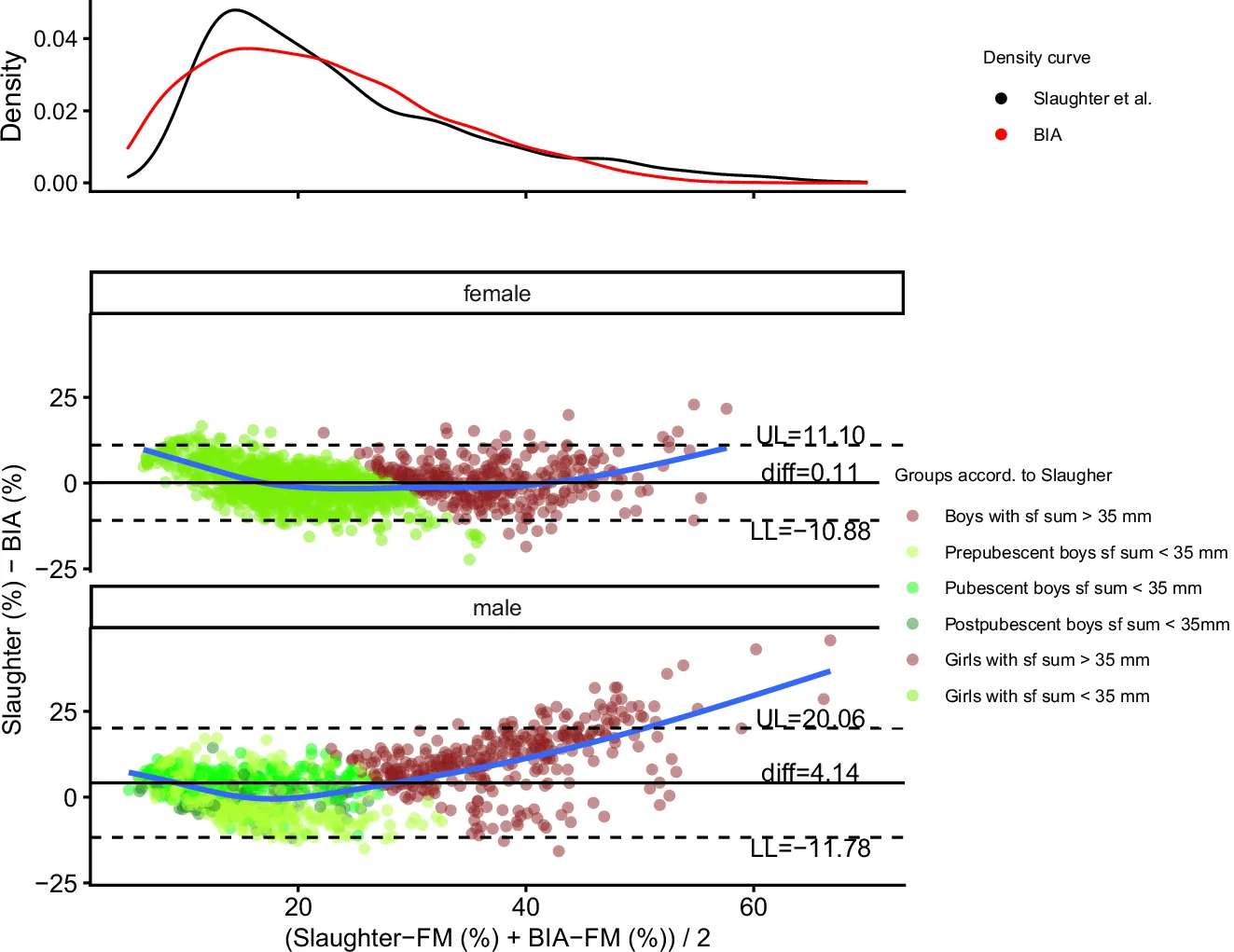
Bland-Altman analyses for FM (%) between skinfold-based Slaughter et al. equations and BIACORPUS RX 4000. The density graph at the top shows the distribution of measurements. Points are colored according to the estimated FM derived from equations by Slaughter et al. Boys with a skinfold sum >35 mm showed a considerable bias to higher skinfold-based estimates when compared with the BIA-based estimates (bias: 4.14). No such difference could be observed for girls (bias: 0.11).

For boys, the Slaughter formulae estimated higher FM (%) than BIA, indicating a systematic bias. The variance and bias increased with increasing FM, suggesting proportional bias. For girls, there was a similar tendency but without discernible systematic or proportionate biases (Fig. 5, Supplementary Table 13).

Age-stratified Bland-Altman analyses showed small but statistically significant differences for most age groups (*p* < 0.001), between BIACORPUS and Slaughter et al. In younger children (<9 years), BIACORPUS-based FM was lower than Slaughter estimates, whereas in preadolescents and adolescents (9–16 years), estimates were slightly higher, with no consistent age trend. In contrast, BIACORPUS-based FM was higher than the Gätjens-based equations across all age groups, without age-related variation (Supplementary Table 14).

## Discussion

### Reliability of segmental BIA measurements

The OCCCs, CCCs, and ICCs showed “good” to “excellent” agreement between the various measurement segments. The agreement improved considerably after excluding RALA and RFLF. Symmetric segments showed no systematic or proportional biases for Rz/H (SDS). Hence, averaging symmetrical derivatives could reduce the influence of measurement errors and produce more stable results. Significant differences were seen in Xc/H-SDS values for girls and the RARF/LALF-Xc/H-SDS values for boys. Depending on the BIA device and measurement protocol, there were contradictory statements regarding the comparability of the segments. Lafontant et al. recently reported differences between the body halves for Rz and Xc [30].

### Age- and sex-specific reference curves for Rz/H and Xc/H

Boys’ Rz/H levels dropped more during puberty than girls’, after which boys’ percentiles remained lower, likely due to greater pubertal muscle growth [31]. Due to the high water content of muscular tissue [32], Rz decreases as muscle mass/FFM increase [15], suggesting that the increase in FFM has stalled [33]. A similar plateau was observed in the Xc/H percentiles, which relates to cell mass and intracellular water [34]. Wells et al. found comparable patterns in a smaller study sample [35]. The use of the raw BIA measures for clinical use is currently subject of ongoing research [15].

### Clinical associations with BMI-SDS

We found lower Rz/H and Xc/H SDS in children and adolescents with obesity. Furthermore, our longitudinal analysis showed that an increase in BMI-SDS was associated with decreasing Rz/H-SDS and Xc/H-SDS.

Prior studies also reported lower Rz and Xc in obesity [23, 24]. Numerous factors, such as increased FM, increased height or muscle mass, and possible hyperhydration in children with obesity may explain the differences. However, animal studies have demonstrated that obesity does not necessarily result in increased muscle mass [36]. Plus, the faster early growth of children with obesity is offset by slower pubertal growth, resulting in similar adult height [37]. Possible hyperhydration mechanisms include hormonal changes (antidiuretic hormone and aldosterone) and adipose tissue inflammation [38, 39].

### Cross-method comparison of different estimation methods for body composition

We compared FM outputs from the proprietary BIACORPUS RX 4000 and the Gätjens equation because both are derived from phase-sensitive SF-BIA devices at a fixed frequency at 50 kHz. Still, there was insufficient agreement between FM estimates. Thus, a critical perspective should be applied when comparing the outputs of various BIA devices [40]. To combat this issue, it may be possible to develop conversion formulas, providing comparable values for, e.g., resistance [41].

When comparing the BIA-based and skinfold-based (Slaughter et al.) estimates of body fat in girls and normal-weight boys, there were no significant discrepancies. But Forte et al. showed that even in normal weight children, the lack of agreement indicates that the methods are not interchangeable [42]. In boys, with skinfold sums above 35 mm or BMI-SDS > 1.881, we found a systematic bias, with lower BIA-based fat mass estimates. The bias increased with increasing estimated FM (see Fig. 5). The underestimation of FM by BIA for participants with obesity has previously been described [6, 43, 44] with varying possible causes [44–47].

While the skinfold-based formulas proposed by Slaughter are widely recommended for estimating body fat in pediatric studies, they warrant critical evaluation [43]. Slaughter et al. originally validated their results using mean values from the literature during the 1980s, before the obesity epidemic [18]. Thus, the measurements used to derive the formulas lack enough cases of severe obesity. Further, equations by Slaughter et al. overestimate or underestimate body fat depending on the reference methods [48]. Regarding the estimates of the BIA algorithms, they may suffer from overfitting to the original study cohorts (Supplementary Fig. 7). In addition to the potential inaccuracies inherent in the equations, several factors can influence the accuracy, including differences in skin compressibility, caliper pressure, device differences (e.g., Harpenden and Holtain calipers), measurement technique, and inter-examiner variation [49].

Age-stratified analyses suggest that the differences between BIACORPUS and Slaughter are not primarily age-driven but may reflect factors like pubertal stage or total body fat. The bias between BIACORPUS and Gätjens et al. likely results from algorithm calibrations rather than biological variation.

### Strengths and limitations of this study

Strengths include the large number of participants, standardized assessments, a broad age range, and longitudinal data. To date, only a limited number of studies have assessed raw bioimpedance measurements, specifically Rz and Xc, and their associations with weight status in children and adolescents. The use of a segmental, phase-sensitive SF-BIA allows detailed evaluation of segmental agreement but may introduce limitations [6, 14]. Single-frequency (50 kHz) systems primarily reflect total body water and provide less tissue characterization than multi-frequency or bioimpedance spectroscopy methods [50]. Their accuracy depends on hydration status, electrode placement, posture, and body geometry. This emphasizes the importance of strict standardization. Device-specific, proprietary algorithms limit comparability across different BIA devices.

No gold standard method such as DXA or air-displacement plethysmography could be applied in this study, in part due to ethical restrictions in Germany. Therefore, a direct validation of measurements for FM and FFM was not possible. As the study population was almost exclusively Caucasian, generalizability is limited and the applicability of the Slaughter et al. equations outside the validated age range (8–17 years) requires caution. Two caliper types were used, which are not fully interchangeable; therefore, each participant was measured with only one caliper type, using Harpenden calipers for higher skinfold thicknesses. To reduce inter-observer variation, all examiners received standardized training and refresher courses. As consecutive repeated measurements were unavailable, data on inter-operator variability could not be quantified.

## Conclusion

The segmental BIA sections appear to be well reproducible, especially with parallel electrode placement. The estimated Rz/H and Xc/H reference values are strongly influenced by age and BMI-SDS.

Body fat estimates show generally good agreement across methods in normal-weight children, whereas accuracy decreases with increasing fat mass. Therefore, current estimation approaches may be unsuitable for children with obesity.

These results provide comprehensive references values for Rz/H and Xc/H from childhood to adolescence. They also identify specific segmental measurements that reduce concordance. BIA-derived body-composition estimates differ from skinfold-based methods based on weight status. This supports the continued use of segmental BIA in normal-weight children with appropriate segment selection and age-/BMI-specific reference values. However, caution is warranted in children with moderate-to-severe obesity.

## Supplementary information

Supplemental Material

## Data Availability

The data set presented in this article cannot be shared publicly due to ethical and legal restrictions. The LIFE Child Study collects potentially sensitive information. Publishing the data is not covered by the informed consent provided by the study participants. Additionally, the LIFE Data Protection Concept requires all (external and internal) researchers who want to access the data to sign a project agreement. Researchers interested in accessing data from the LIFE Child Study may contact the study by writing to forschungsdaten@medizin.uni-leipzig.de.
