# Supplementary material for: Segmental BIA-derived body composition in children and adolescents aged 5–18 years: age- and sex-specific reference values and limitations in obesity
Source: Eur J Clin Nutr. 2026 Aug 1;80(10):1062–9. doi: 10.1038/s41430-026-01788-1 (PMC13690603; doi:10.1038/s41430-026-01788-1)
Supplement: Supplementary file 1 [file 41430_2026_1788_MOESM1_ESM.docx]

## Supplement

**Supplementary material for Böker et al. *„Segmental BIA-derived body composition in children and adolescents aged 5 – 18 years: age- and sex-specific reference values and limitations in obesity”.***

For Triceps and Subscapular:

Prepubescent White Males: PFDWB =1.21 * (triceps + subscapular) - .008 (triceps + subscapular)^2^ - 1.7

Pubescent White Males: PFDWB =1.21 (triceps + subscapular) - .008 (triceps + subscapular)^2^ - 3.4

Postpubescent White Males: PFDWB =1.21 (triceps + subscapular) - .008 (triceps + subscapular)^2^ - 5.5

All Females: PFDWB = 1.33 (triceps + subscapular) - .013 (triceps + subscapular)^2^ - 2.5

For a sum of tricep and subscapular greater than 35 mm:

All Males PFDWB = .783 (triceps + subscapular) + 1.6

All Females PFDWB = .546 (triceps + subscapular) + 9.7

**Skinfold thickness formulas** used to calculate fat mass in children and adolescents from “Skinfold Equations for Estimation of Body Fatness in Children and Youth” by Slaughter et al.

FFM [kg] = 0.66 × [height^2^ (cm^2^)/resistance (Ω)] + 0.196 × weight [kg] + 0.157 × reactance [Ω] + 0.348 × age [years] − 12,083

FM [kg] = weight [kg] - FFM [kg]

**Body composition formulas** used to calculate fat mass and fat-free mass in children and adolescents from “Body Composition Characteristics of a Load-Capacity Model: Age-Dependent and Sex-Specific Percentiles in 5- to 17-Year-Old Children” by Gätjens et al.

**Supplementary Figure 1:** The study sample comprised all children between the ages of 4 and 21 years with at least one bioimpedance measurement and one triceps and subscapular fold measurement. Children with relevant medical conditions or implausible measurements were excluded.

**Anthropometric Measures:** Body height was measured with a “Dr. Keller I” stadiometer (Längenmesstechnik GmbH Limbach, Limbach-Oberfrohna, Germany) with an accuracy of 0.1 cm. Weight was measured with children wearing light underclothes and without shoes using a “Seca 704” electronic scale (Seca GmbH and Co. KG, Hamburg, Germany) with an accuracy of 50 g up to 150 kg and an accuracy of 100 g for weight measuring up to 300 kg (n = 7). The subscapular and triceps skinfold was measured 3 times on the right body side in a standing position using a “Holtain” (Holtain, Crosswell) or “Harpenden Skinfold” (Baty International, Burgess Hill) caliper, both with an accuracy of 0.2 mm, and averaged (see Rönnecke E, Vogel M, Bussler S, Grafe N, Jurkutat A, Schlingmann M, et al. Age- and sex-related percentiles of skinfold thickness, waist and hip circumference, waist-to-hip ratio and waist-to-height ratio: results from a population-based pediatric cohort in Germany (LIFE Child). Obes Facts. 2019;12(1):25–39.).

**Supplementary Figure 2:** showing the electrode configuration and body segments to aid interpretation of the segmental analyses using the BodyMapR package (Miller DM, Shalhout SZ. BodyMapR: An R package and Shiny application for creating anatomical visualizations of cancer lesions. JAMIA Open. 2022;5(1):ooac013. doi:10.1093/jamiaopen/ooac013.) and PowerPoint.

**Supplementary Figure 3**: We found the highest CCCs between the arm-leg leads representing the body halves (RARF and LALF), while the horizontal derivatives RFLF and RALA had significantly lower CCCs than the other derivatives. Abbreviations: right (r), left (l), arm (a) and foot (f).

**Supplementary Figure 4**: Results of Bland-Altman analyses comparing the Rz/H-SDS (A) and Xc/H-SDS (B) for RALF and LARF. No significant systematic differences were found for Rz/H (bias girls: 0.02, bias boys: 0.001). Slight systemic bias in the Xc/H-SDS could be found for girls but not for boys (bias girls: -0.05, bias boys: -0.01).

**Supplementary Figure 5**: Compared with the reference ranges derived from a non-obese study population, Xc/H values were lower for children and adolescents with obesity or severe obesity, with most values below the 50th percentile. The other derivatives showed similar patterns.

**Supplementary Figure 6**: Bland-Altman analyses for the FFM kg (A) and FFM % (B) comparing estimates of BIACORPUS RX 4000 and Gätjens et al. The BIACORPUS algorithm estimated higher FFM for both sexes (bias FFM (kg) girls: 0.11, FFM (kg) boys: 1.50; bias FFM (%) girls: 0.01, FFM (%) boys: 0.03).

**Supplementary Figure 7**: Bland-Altman analyses stratified by FFM (kg) intervals. The degree of agreement for the FFM (kg) varied by mean FFM.

**Supplementary Figure 8**: Bland-Altman analyses for the FM kg (A) and FM % (B) between BIACORPUS RX 4000 and Gätjens et al. BIACORPUS algorithm estimated lower values for FM than Gätjens et al. (bias FM (kg) girls: -0.13, FM (kg) boys: - 1.5; bias FM (%) girls: - 0.01, FM (%) boys: -0.03)

| sex | age | perc_02_5 | perc_50_0 | perc_97_5 | mu | sigma | nu | tau |
| --- | --- | --- | --- | --- | --- | --- | --- | --- |
| female | 5 | 5.89312748 | 7.04464887 | 8.40715607 | 7.0446 | 0.0889 | 0.0528 | 1.7167 |
| female | 5.5 | 5.631654 | 6.76167394 | 8.1042512 | 6.7617 | 0.091 | 0.0528 | 1.7167 |
| female | 6 | 5.38202362 | 6.48923364 | 7.80991679 | 6.4892 | 0.0931 | 0.0528 | 1.7167 |
| female | 6.5 | 5.15136089 | 6.23611872 | 7.53490239 | 6.2361 | 0.0951 | 0.0528 | 1.7167 |
| female | 7 | 4.94922271 | 6.0142221 | 7.29389755 | 6.0142 | 0.097 | 0.0528 | 1.7167 |
| female | 7.5 | 4.77770582 | 5.82650575 | 7.09092771 | 5.8265 | 0.0988 | 0.0528 | 1.7167 |
| female | 8 | 4.62719559 | 5.66161533 | 6.91256355 | 5.6616 | 0.1004 | 0.0528 | 1.7167 |
| female | 8.5 | 4.48709957 | 5.50682198 | 6.74349294 | 5.5068 | 0.1019 | 0.0528 | 1.7167 |
| female | 9 | 4.34796322 | 5.35063602 | 6.56973917 | 5.3506 | 0.1032 | 0.0528 | 1.7167 |
| female | 9.5 | 4.20023543 | 5.18133604 | 6.37691045 | 5.1813 | 0.1044 | 0.0528 | 1.7167 |
| female | 10 | 4.03526529 | 4.98823494 | 6.15180091 | 4.9882 | 0.1055 | 0.0528 | 1.7167 |
| female | 10.5 | 3.85200859 | 4.77003348 | 5.89276853 | 4.77 | 0.1063 | 0.0528 | 1.7167 |
| female | 11 | 3.66560705 | 4.54556259 | 5.62314937 | 4.5456 | 0.107 | 0.0528 | 1.7167 |
| female | 11.5 | 3.49843741 | 4.34278201 | 5.37776805 | 4.3428 | 0.1075 | 0.0528 | 1.7167 |
| female | 12 | 3.36578261 | 4.18094307 | 5.18078949 | 4.1809 | 0.1079 | 0.0528 | 1.7167 |
| female | 12.5 | 3.27067045 | 4.06405726 | 5.03747971 | 4.0641 | 0.108 | 0.0528 | 1.7167 |
| female | 13 | 3.20901746 | 3.98721862 | 4.94195793 | 3.9872 | 0.108 | 0.0528 | 1.7167 |
| female | 13.5 | 3.17291692 | 3.94069018 | 4.88226177 | 3.9407 | 0.1078 | 0.0528 | 1.7167 |
| female | 14 | 3.15250726 | 3.91225919 | 4.84330707 | 3.9123 | 0.1074 | 0.0528 | 1.7167 |
| female | 14.5 | 3.13696072 | 3.88851249 | 4.80852518 | 3.8885 | 0.1068 | 0.0528 | 1.7167 |
| female | 15 | 3.12146272 | 3.86352797 | 4.77065886 | 3.8635 | 0.1061 | 0.0528 | 1.7167 |
| female | 15.5 | 3.10892202 | 3.84096548 | 4.73431243 | 3.841 | 0.1052 | 0.0528 | 1.7167 |
| female | 16 | 3.10636071 | 3.82953253 | 4.71027266 | 3.8295 | 0.1041 | 0.0528 | 1.7167 |
| female | 16.5 | 3.11723592 | 3.83346738 | 4.70374149 | 3.8335 | 0.1029 | 0.0528 | 1.7167 |
| female | 17 | 3.13895246 | 3.8495237 | 4.71068917 | 3.8495 | 0.1015 | 0.0528 | 1.7167 |
| female | 17.5 | 3.1649803 | 3.86966217 | 4.72126175 | 3.8697 | 0.1 | 0.0528 | 1.7167 |
| female | 18 | 3.18969257 | 3.88703566 | 4.72716651 | 3.887 | 0.0984 | 0.0528 | 1.7167 |
| male | 5 | 5.74784569 | 6.64029622 | 7.65178687 | 6.6403 | 0.0725 | 0.1246 | 1.8921 |
| male | 5.5 | 5.49639085 | 6.41702519 | 7.46994044 | 6.417 | 0.0777 | 0.1246 | 1.8921 |
| male | 6 | 5.25933317 | 6.20523044 | 7.29684932 | 6.2052 | 0.0829 | 0.1246 | 1.8921 |
| male | 6.5 | 5.03322345 | 6.00059446 | 7.12699419 | 6.0006 | 0.0881 | 0.1246 | 1.8921 |
| male | 7 | 4.81669791 | 5.80119823 | 6.95756726 | 5.8012 | 0.0931 | 0.1246 | 1.8921 |
| male | 7.5 | 4.61327354 | 5.61105985 | 6.79291356 | 5.6111 | 0.098 | 0.1246 | 1.8921 |
| male | 8 | 4.42842128 | 5.43683416 | 6.64084345 | 5.4368 | 0.1026 | 0.1246 | 1.8921 |
| male | 8.5 | 4.26335575 | 5.28021231 | 6.50339612 | 5.2802 | 0.1069 | 0.1246 | 1.8921 |
| male | 9 | 4.11444093 | 5.13692265 | 6.3753212 | 5.1369 | 0.1109 | 0.1246 | 1.8921 |
| male | 9.5 | 3.97367776 | 4.99710058 | 6.24426705 | 4.9971 | 0.1144 | 0.1246 | 1.8921 |
| male | 10 | 3.83231873 | 4.84971396 | 6.09617203 | 4.8497 | 0.1175 | 0.1246 | 1.8921 |
| male | 10.5 | 3.68691864 | 4.69032348 | 5.92514396 | 4.6903 | 0.1201 | 0.1246 | 1.8921 |
| male | 11 | 3.53732234 | 4.51876909 | 5.73083675 | 4.5188 | 0.1222 | 0.1246 | 1.8921 |
| male | 11.5 | 3.38310006 | 4.33471839 | 5.51293678 | 4.3347 | 0.1236 | 0.1246 | 1.8921 |
| male | 12 | 3.22348219 | 4.13758048 | 5.27105826 | 4.1376 | 0.1245 | 0.1246 | 1.8921 |
| male | 12.5 | 3.06043148 | 3.93044805 | 5.0097664 | 3.9304 | 0.1248 | 0.1246 | 1.8921 |
| male | 13 | 2.90173107 | 3.72404395 | 4.74358178 | 3.724 | 0.1244 | 0.1246 | 1.8921 |
| male | 13.5 | 2.75841595 | 3.53330476 | 4.4924771 | 3.5333 | 0.1235 | 0.1246 | 1.8921 |
| male | 14 | 2.64025391 | 3.37139784 | 4.27402688 | 3.3714 | 0.122 | 0.1246 | 1.8921 |
| male | 14.5 | 2.55056991 | 3.24298936 | 4.09471726 | 3.243 | 0.1199 | 0.1246 | 1.8921 |
| male | 15 | 2.48457668 | 3.14220684 | 3.94747138 | 3.1422 | 0.1172 | 0.1246 | 1.8921 |
| male | 15.5 | 2.43456685 | 3.0594535 | 3.82050276 | 3.0595 | 0.1141 | 0.1246 | 1.8921 |
| male | 16 | 2.3947204 | 2.98760831 | 3.70524125 | 2.9876 | 0.1105 | 0.1246 | 1.8921 |
| male | 16.5 | 2.36239758 | 2.92362144 | 3.59828253 | 2.9236 | 0.1065 | 0.1246 | 1.8921 |
| male | 17 | 2.33470469 | 2.86418509 | 3.49595891 | 2.8642 | 0.1022 | 0.1246 | 1.8921 |
| male | 17.5 | 2.30949781 | 2.80698805 | 3.39589107 | 2.807 | 0.0976 | 0.1246 | 1.8921 |
| male | 18 | 2.28605866 | 2.75149936 | 3.29788621 | 2.7515 | 0.0928 | 0.1246 | 1.8921 |

**Supplementary Table 1:** Percentile table of the derivative right-arm-right-foot (RARF) for Rz/H in ohm/cm

| sex | age | perc_02_5 | perc_50_0 | perc_97_5 | mu | sigma | nu | tau |
| --- | --- | --- | --- | --- | --- | --- | --- | --- |
| female | 5 | 6.11771336 | 7.33650679 | 8.69409741 | 7.3365 | 0.0873 | 0.3854 | 1.6407 |
| female | 5.5 | 5.86335971 | 7.08406214 | 8.44959082 | 7.0841 | 0.0907 | 0.3854 | 1.6407 |
| female | 6 | 5.61920002 | 6.83892433 | 8.20906957 | 6.8389 | 0.0941 | 0.3854 | 1.6407 |
| female | 6.5 | 5.3893387 | 6.60614275 | 7.97857185 | 6.6061 | 0.0974 | 0.3854 | 1.6407 |
| female | 7 | 5.17962221 | 6.39309078 | 7.76713694 | 6.3931 | 0.1006 | 0.3854 | 1.6407 |
| female | 7.5 | 4.99181101 | 6.20228485 | 7.57809863 | 6.2023 | 0.1036 | 0.3854 | 1.6407 |
| female | 8 | 4.82063374 | 6.02751687 | 7.40414861 | 6.0275 | 0.1065 | 0.3854 | 1.6407 |
| female | 8.5 | 4.65867417 | 5.85972396 | 7.23429759 | 5.8597 | 0.1092 | 0.3854 | 1.6407 |
| female | 9 | 4.49741596 | 5.68825083 | 7.05537754 | 5.6883 | 0.1117 | 0.3854 | 1.6407 |
| female | 9.5 | 4.328254 | 5.50213337 | 6.85363629 | 5.5021 | 0.114 | 0.3854 | 1.6407 |
| female | 10 | 4.14526723 | 5.29368577 | 6.61926914 | 5.2937 | 0.1161 | 0.3854 | 1.6407 |
| female | 10.5 | 3.95063935 | 5.06557838 | 6.35543121 | 5.0656 | 0.1179 | 0.3854 | 1.6407 |
| female | 11 | 3.75859233 | 4.83612645 | 6.0851306 | 4.8361 | 0.1195 | 0.3854 | 1.6407 |
| female | 11.5 | 3.58756886 | 4.62941495 | 5.83899789 | 4.6294 | 0.1208 | 0.3854 | 1.6407 |
| female | 12 | 3.44969592 | 4.46162302 | 5.63795778 | 4.4616 | 0.1218 | 0.3854 | 1.6407 |
| female | 12.5 | 3.34723136 | 4.33619836 | 5.48689185 | 4.3362 | 0.1225 | 0.3854 | 1.6407 |
| female | 13 | 3.27668763 | 4.24903006 | 5.38100599 | 4.249 | 0.123 | 0.3854 | 1.6407 |
| female | 13.5 | 3.23114175 | 4.19140365 | 5.30952877 | 4.1914 | 0.1231 | 0.3854 | 1.6407 |
| female | 14 | 3.20182817 | 4.15209691 | 5.25839606 | 4.1521 | 0.123 | 0.3854 | 1.6407 |
| female | 14.5 | 3.18044411 | 4.12043375 | 5.21418509 | 4.1204 | 0.1226 | 0.3854 | 1.6407 |
| female | 15 | 3.16449251 | 4.09326257 | 5.17300383 | 4.0933 | 0.1219 | 0.3854 | 1.6407 |
| female | 15.5 | 3.15657973 | 4.07403394 | 5.13931012 | 4.074 | 0.1209 | 0.3854 | 1.6407 |
| female | 16 | 3.16005047 | 4.06710893 | 5.1186706 | 4.0671 | 0.1196 | 0.3854 | 1.6407 |
| female | 16.5 | 3.17342538 | 4.07058841 | 5.10872351 | 4.0706 | 0.1181 | 0.3854 | 1.6407 |
| female | 17 | 3.19095895 | 4.07712445 | 5.10029685 | 4.0771 | 0.1163 | 0.3854 | 1.6407 |
| female | 17.5 | 3.20669873 | 4.07921568 | 5.08414533 | 4.0792 | 0.1143 | 0.3854 | 1.6407 |
| female | 18 | 3.21852354 | 4.07436029 | 5.05738756 | 4.0744 | 0.1121 | 0.3854 | 1.6407 |
| male | 5 | 5.98121496 | 6.96488345 | 8.14423227 | 6.9649 | 0.0778 | -0.1752 | 1.832 |
| male | 5.5 | 5.75251638 | 6.76451889 | 7.99230632 | 6.7645 | 0.0829 | -0.1752 | 1.832 |
| male | 6 | 5.53502038 | 6.57283909 | 7.84699124 | 6.5728 | 0.088 | -0.1752 | 1.832 |
| male | 6.5 | 5.31983791 | 6.3790009 | 7.69479893 | 6.379 | 0.0931 | -0.1752 | 1.832 |
| male | 7 | 5.10505703 | 6.18023562 | 7.5315551 | 6.1802 | 0.098 | -0.1752 | 1.832 |
| male | 7.5 | 4.8978896 | 5.98488771 | 7.36665735 | 5.9849 | 0.1029 | -0.1752 | 1.832 |
| male | 8 | 4.70763719 | 5.80426289 | 7.21363642 | 5.8043 | 0.1076 | -0.1752 | 1.832 |
| male | 8.5 | 4.538352 | 5.64359569 | 7.07898797 | 5.6436 | 0.1121 | -0.1752 | 1.832 |
| male | 9 | 4.38656311 | 5.49888889 | 6.95779234 | 5.4989 | 0.1163 | -0.1752 | 1.832 |
| male | 9.5 | 4.24170779 | 5.3570698 | 6.83340124 | 5.3571 | 0.1202 | -0.1752 | 1.832 |
| male | 10 | 4.09320082 | 5.20468668 | 6.68820685 | 5.2047 | 0.1238 | -0.1752 | 1.832 |
| male | 10.5 | 3.93937953 | 5.03943319 | 6.51865209 | 5.0394 | 0.1269 | -0.1752 | 1.832 |
| male | 11 | 3.77981549 | 4.86066446 | 6.32345976 | 4.8607 | 0.1297 | -0.1752 | 1.832 |
| male | 11.5 | 3.60991014 | 4.6624759 | 6.09468544 | 4.6625 | 0.132 | -0.1752 | 1.832 |
| male | 12 | 3.42381084 | 4.43739833 | 5.82246055 | 4.4374 | 0.1338 | -0.1752 | 1.832 |
| male | 12.5 | 3.22206266 | 4.18637529 | 5.50818056 | 4.1864 | 0.1351 | -0.1752 | 1.832 |
| male | 13 | 3.01683607 | 3.92570017 | 5.17384829 | 3.9257 | 0.1359 | -0.1752 | 1.832 |
| male | 13.5 | 2.82634668 | 3.67977665 | 4.85256666 | 3.6798 | 0.1362 | -0.1752 | 1.832 |
| male | 14 | 2.66694125 | 3.47062955 | 4.57443154 | 3.4706 | 0.136 | -0.1752 | 1.832 |
| male | 14.5 | 2.54554535 | 3.30785552 | 4.35295439 | 3.3079 | 0.1352 | -0.1752 | 1.832 |
| male | 15 | 2.45658131 | 3.18455515 | 4.17960792 | 3.1846 | 0.1339 | -0.1752 | 1.832 |
| male | 15.5 | 2.3896993 | 3.08750491 | 4.03738726 | 3.0875 | 0.1322 | -0.1752 | 1.832 |
| male | 16 | 2.33656886 | 3.00609632 | 3.91273187 | 3.0061 | 0.1299 | -0.1752 | 1.832 |
| male | 16.5 | 2.29256189 | 2.93454555 | 3.79845897 | 2.9345 | 0.1273 | -0.1752 | 1.832 |
| male | 17 | 2.25317892 | 2.86729674 | 3.68777358 | 2.8673 | 0.1242 | -0.1752 | 1.832 |
| male | 17.5 | 2.21575216 | 2.80121406 | 3.57711386 | 2.8012 | 0.1207 | -0.1752 | 1.832 |
| male | 18 | 2.18008105 | 2.73633189 | 3.46702447 | 2.7363 | 0.1169 | -0.1752 | 1.832 |

**Supplementary Table 2:** Percentile table of the derivative right-arm-left-foot (RALA) for Rz/H in ohm/cm

| sex | age | perc_02_5 | perc_50_0 | perc_97_5 | mu | sigma | nu | tau |
| --- | --- | --- | --- | --- | --- | --- | --- | --- |
| female | 5 | 4.7977965 | 5.91330924 | 7.31752208 | 5.9133 | 0.1058 | -0.0902 | 1.7414 |
| female | 5.5 | 4.58966224 | 5.67045841 | 7.03462953 | 5.6705 | 0.107 | -0.0902 | 1.7414 |
| female | 6 | 4.3937942 | 5.44088779 | 6.76588541 | 5.4409 | 0.1082 | -0.0902 | 1.7414 |
| female | 6.5 | 4.21714005 | 5.2333416 | 6.52232009 | 5.2333 | 0.1093 | -0.0902 | 1.7414 |
| female | 7 | 4.06496488 | 5.05454375 | 6.31253664 | 5.0545 | 0.1103 | -0.0902 | 1.7414 |
| female | 7.5 | 3.93425354 | 4.90094394 | 6.13233873 | 4.9009 | 0.1112 | -0.0902 | 1.7414 |
| female | 8 | 3.81356297 | 4.75843409 | 5.96424603 | 4.7584 | 0.112 | -0.0902 | 1.7414 |
| female | 8.5 | 3.69487361 | 4.61708403 | 5.79589558 | 4.6171 | 0.1128 | -0.0902 | 1.7414 |
| female | 9 | 3.57421483 | 4.47197582 | 5.62115691 | 4.472 | 0.1134 | -0.0902 | 1.7414 |
| female | 9.5 | 3.44846463 | 4.31924112 | 5.43520502 | 4.3192 | 0.114 | -0.0902 | 1.7414 |
| female | 10 | 3.31439692 | 4.15488095 | 5.23305247 | 4.1549 | 0.1144 | -0.0902 | 1.7414 |
| female | 10.5 | 3.17401686 | 3.98146586 | 5.01800374 | 3.9815 | 0.1148 | -0.0902 | 1.7414 |
| female | 11 | 3.03850429 | 3.81310694 | 4.80795238 | 3.8131 | 0.115 | -0.0902 | 1.7414 |
| female | 11.5 | 2.92094544 | 3.6663384 | 4.62389003 | 3.6663 | 0.1151 | -0.0902 | 1.7414 |
| female | 12 | 2.82804116 | 3.54967795 | 4.4766978 | 3.5497 | 0.1151 | -0.0902 | 1.7414 |
| female | 12.5 | 2.76028419 | 3.46382107 | 4.36735408 | 3.4638 | 0.115 | -0.0902 | 1.7414 |
| female | 13 | 2.71513681 | 3.4056251 | 4.29195307 | 3.4056 | 0.1147 | -0.0902 | 1.7414 |
| female | 13.5 | 2.68773337 | 3.36900113 | 4.24284263 | 3.369 | 0.1144 | -0.0902 | 1.7414 |
| female | 14 | 2.67067916 | 3.34468212 | 4.20835875 | 3.3447 | 0.1139 | -0.0902 | 1.7414 |
| female | 14.5 | 2.65501407 | 3.32145902 | 4.17441933 | 3.3215 | 0.1134 | -0.0902 | 1.7414 |
| female | 15 | 2.63724298 | 3.29499642 | 4.13563028 | 3.295 | 0.1127 | -0.0902 | 1.7414 |
| female | 15.5 | 2.62082902 | 3.26966699 | 4.09754662 | 3.2697 | 0.112 | -0.0902 | 1.7414 |
| female | 16 | 2.61340276 | 3.25501122 | 4.07215907 | 3.255 | 0.1111 | -0.0902 | 1.7414 |
| female | 16.5 | 2.62121397 | 3.25878422 | 4.06913981 | 3.2588 | 0.1102 | -0.0902 | 1.7414 |
| female | 17 | 2.64383683 | 3.28038812 | 4.08766176 | 3.2804 | 0.1092 | -0.0902 | 1.7414 |
| female | 17.5 | 2.6752754 | 3.31231869 | 4.1182993 | 3.3123 | 0.1081 | -0.0902 | 1.7414 |
| female | 18 | 2.7086683 | 3.34605807 | 4.15044241 | 3.3461 | 0.1069 | -0.0902 | 1.7414 |
| male | 5 | 4.7409521 | 5.57707779 | 6.58183177 | 5.5771 | 0.0831 | -0.1197 | 1.8876 |
| male | 5.5 | 4.51287661 | 5.35992124 | 6.38901879 | 5.3599 | 0.088 | -0.1197 | 1.8876 |
| male | 6 | 4.30087945 | 5.15656159 | 6.20744736 | 5.1566 | 0.0929 | -0.1197 | 1.8876 |
| male | 6.5 | 4.10533594 | 4.96757626 | 6.03773838 | 4.9676 | 0.0977 | -0.1197 | 1.8876 |
| male | 7 | 3.92674782 | 4.79376266 | 5.88085111 | 4.7938 | 0.1022 | -0.1197 | 1.8876 |
| male | 7.5 | 3.76572912 | 4.63612047 | 5.73807063 | 4.6361 | 0.1066 | -0.1197 | 1.8876 |
| male | 8 | 3.62212864 | 4.49475742 | 5.60964577 | 4.4948 | 0.1107 | -0.1197 | 1.8876 |
| male | 8.5 | 3.49390654 | 4.36743564 | 5.49290541 | 4.3674 | 0.1145 | -0.1197 | 1.8876 |
| male | 9 | 3.37703173 | 4.24935252 | 5.38186873 | 4.2494 | 0.118 | -0.1197 | 1.8876 |
| male | 9.5 | 3.26557594 | 4.13319157 | 5.26722134 | 4.1332 | 0.121 | -0.1197 | 1.8876 |
| male | 10 | 3.15298047 | 4.01070292 | 5.13829976 | 4.0107 | 0.1236 | -0.1197 | 1.8876 |
| male | 10.5 | 3.03593957 | 3.87770517 | 4.98958535 | 3.8777 | 0.1258 | -0.1197 | 1.8876 |
| male | 11 | 2.91500617 | 3.73500175 | 4.82208268 | 3.735 | 0.1274 | -0.1197 | 1.8876 |
| male | 11.5 | 2.79160602 | 3.5846407 | 4.6386148 | 3.5846 | 0.1285 | -0.1197 | 1.8876 |
| male | 12 | 2.66688508 | 3.4284291 | 4.44189809 | 3.4284 | 0.1291 | -0.1197 | 1.8876 |
| male | 12.5 | 2.54354478 | 3.27026645 | 4.23752982 | 3.2703 | 0.1292 | -0.1197 | 1.8876 |
| male | 13 | 2.42764014 | 3.11841941 | 4.03688026 | 3.1184 | 0.1287 | -0.1197 | 1.8876 |
| male | 13.5 | 2.32683571 | 2.983196 | 3.85395669 | 2.9832 | 0.1277 | -0.1197 | 1.8876 |
| male | 14 | 2.24731303 | 2.87287193 | 3.69998318 | 2.8729 | 0.1262 | -0.1197 | 1.8876 |
| male | 14.5 | 2.19002738 | 2.78885778 | 3.57710549 | 2.7889 | 0.1242 | -0.1197 | 1.8876 |
| male | 15 | 2.15023768 | 2.72518877 | 3.47785541 | 2.7252 | 0.1217 | -0.1197 | 1.8876 |
| male | 15.5 | 2.12155359 | 2.67382357 | 3.39214185 | 2.6738 | 0.1188 | -0.1197 | 1.8876 |
| male | 16 | 2.0994482 | 2.62916538 | 3.31310808 | 2.6292 | 0.1155 | -0.1197 | 1.8876 |
| male | 16.5 | 2.08212345 | 2.58911864 | 3.23841882 | 2.5891 | 0.1118 | -0.1197 | 1.8876 |
| male | 17 | 2.06813753 | 2.55206839 | 3.16638073 | 2.5521 | 0.1078 | -0.1197 | 1.8876 |
| male | 17.5 | 2.05655791 | 2.51706116 | 3.09615043 | 2.5171 | 0.1036 | -0.1197 | 1.8876 |
| male | 18 | 2.04693905 | 2.48375748 | 3.02764476 | 2.4838 | 0.0991 | -0.1197 | 1.8876 |

**Supplementary Table 3:** Percentile table of the derivative right-foot-left-foot (RFLF) for Rz/H in ohm/cm

| sex | age | perc_02_5 | perc_50_0 | perc_97_5 | mu | sigma | nu | tau |
| --- | --- | --- | --- | --- | --- | --- | --- | --- |
| female | 5 | 5.70444978 | 6.87098915 | 8.1890392 | 6.871 | 0.0898 | 0.3237 | 1.6434 |
| female | 5.5 | 5.46490591 | 6.6175182 | 7.92424053 | 6.6175 | 0.0923 | 0.3237 | 1.6434 |
| female | 6 | 5.23788529 | 6.37523867 | 7.66886974 | 6.3752 | 0.0947 | 0.3237 | 1.6434 |
| female | 6.5 | 5.0288702 | 6.15101517 | 7.43133428 | 6.151 | 0.097 | 0.3237 | 1.6434 |
| female | 7 | 4.8420978 | 5.95032545 | 7.21851403 | 5.9503 | 0.0992 | 0.3237 | 1.6434 |
| female | 7.5 | 4.67602379 | 5.77160804 | 7.02882389 | 5.7716 | 0.1012 | 0.3237 | 1.6434 |
| female | 8 | 4.52341274 | 5.60620765 | 6.8519714 | 5.6062 | 0.1031 | 0.3237 | 1.6434 |
| female | 8.5 | 4.37790198 | 5.4464066 | 6.67866128 | 5.4464 | 0.1049 | 0.3237 | 1.6434 |
| female | 9 | 4.23350378 | 5.28484601 | 6.4999275 | 5.2848 | 0.1065 | 0.3237 | 1.6434 |
| female | 9.5 | 4.08319755 | 5.11279532 | 6.30503277 | 5.1128 | 0.1079 | 0.3237 | 1.6434 |
| female | 10 | 3.92070703 | 4.9223933 | 6.08424868 | 4.9224 | 0.1091 | 0.3237 | 1.6434 |
| female | 10.5 | 3.74756675 | 4.71558569 | 5.83997331 | 4.7156 | 0.1102 | 0.3237 | 1.6434 |
| female | 11 | 3.57703655 | 4.50918293 | 5.59313718 | 4.5092 | 0.111 | 0.3237 | 1.6434 |
| female | 11.5 | 3.42588619 | 4.32458339 | 5.37054221 | 4.3246 | 0.1116 | 0.3237 | 1.6434 |
| female | 12 | 3.30422869 | 4.17487218 | 5.18876825 | 4.1749 | 0.1121 | 0.3237 | 1.6434 |
| female | 12.5 | 3.21383674 | 4.06257334 | 5.05124955 | 4.0626 | 0.1123 | 0.3237 | 1.6434 |
| female | 13 | 3.15187718 | 3.98430703 | 4.95399639 | 3.9843 | 0.1123 | 0.3237 | 1.6434 |
| female | 13.5 | 3.11209872 | 3.93228976 | 4.8874584 | 3.9323 | 0.1121 | 0.3237 | 1.6434 |
| female | 14 | 3.0857202 | 3.89549002 | 4.83799626 | 3.8955 | 0.1117 | 0.3237 | 1.6434 |
| female | 14.5 | 3.06449591 | 3.86354945 | 4.79280666 | 3.8635 | 0.111 | 0.3237 | 1.6434 |
| female | 15 | 3.04643105 | 3.83401885 | 4.74893076 | 3.834 | 0.1102 | 0.3237 | 1.6434 |
| female | 15.5 | 3.03497677 | 3.81130534 | 4.7119071 | 3.8113 | 0.1092 | 0.3237 | 1.6434 |
| female | 16 | 3.03478347 | 3.80127464 | 4.68902847 | 3.8013 | 0.108 | 0.3237 | 1.6434 |
| female | 16.5 | 3.04682873 | 3.80512742 | 4.68176565 | 3.8051 | 0.1067 | 0.3237 | 1.6434 |
| female | 17 | 3.06653481 | 3.81711207 | 4.68302169 | 3.8171 | 0.1051 | 0.3237 | 1.6434 |
| female | 17.5 | 3.0869338 | 3.82857378 | 4.68221641 | 3.8286 | 0.1035 | 0.3237 | 1.6434 |
| female | 18 | 3.10372121 | 3.83427507 | 4.67307153 | 3.8343 | 0.1016 | 0.3237 | 1.6434 |
| male | 5 | 5.64047128 | 6.53843902 | 7.58804222 | 6.5384 | 0.0754 | -0.052 | 1.9479 |
| male | 5.5 | 5.39070825 | 6.31082063 | 7.39761378 | 6.3108 | 0.0805 | -0.052 | 1.9479 |
| male | 6 | 5.15660134 | 6.09623982 | 7.21770737 | 6.0962 | 0.0855 | -0.052 | 1.9479 |
| male | 6.5 | 4.93474716 | 5.89058247 | 7.0431432 | 5.8906 | 0.0904 | -0.052 | 1.9479 |
| male | 7 | 4.72620979 | 5.69501682 | 6.8749638 | 5.695 | 0.0953 | -0.052 | 1.9479 |
| male | 7.5 | 4.53519371 | 5.5146352 | 6.71909216 | 5.5146 | 0.0999 | -0.052 | 1.9479 |
| male | 8 | 4.36445132 | 5.35298384 | 6.5798228 | 5.353 | 0.1044 | -0.052 | 1.9479 |
| male | 8.5 | 4.21386578 | 5.21021231 | 6.45742539 | 5.2102 | 0.1085 | -0.052 | 1.9479 |
| male | 9 | 4.0785706 | 5.08056937 | 6.34482813 | 5.0806 | 0.1123 | -0.052 | 1.9479 |
| male | 9.5 | 3.94896719 | 4.95221205 | 6.22711348 | 4.9522 | 0.1158 | -0.052 | 1.9479 |
| male | 10 | 3.81539541 | 4.8129539 | 6.08860509 | 4.813 | 0.1188 | -0.052 | 1.9479 |
| male | 10.5 | 3.67563941 | 4.65987242 | 5.92520098 | 4.6599 | 0.1214 | -0.052 | 1.9479 |
| male | 11 | 3.53086795 | 4.49444575 | 5.73856405 | 4.4944 | 0.1235 | -0.052 | 1.9479 |
| male | 11.5 | 3.38016682 | 4.31566939 | 5.5274459 | 4.3157 | 0.125 | -0.052 | 1.9479 |
| male | 12 | 3.22072986 | 4.12025925 | 5.28790295 | 4.1203 | 0.126 | -0.052 | 1.9479 |
| male | 12.5 | 3.0535218 | 3.90989845 | 5.02260403 | 3.9099 | 0.1265 | -0.052 | 1.9479 |
| male | 13 | 2.88778646 | 3.6970324 | 4.74830327 | 3.697 | 0.1264 | -0.052 | 1.9479 |
| male | 13.5 | 2.73718882 | 3.4998398 | 4.48925569 | 3.4998 | 0.1258 | -0.052 | 1.9479 |
| male | 14 | 2.61384104 | 3.33439982 | 4.26690393 | 3.3344 | 0.1246 | -0.052 | 1.9479 |
| male | 14.5 | 2.52226933 | 3.20685601 | 4.08965763 | 3.2069 | 0.1229 | -0.052 | 1.9479 |
| male | 15 | 2.45705668 | 3.11048018 | 3.94922489 | 3.1105 | 0.1206 | -0.052 | 1.9479 |
| male | 15.5 | 2.40885225 | 3.03352194 | 3.83089319 | 3.0335 | 0.1179 | -0.052 | 1.9479 |
| male | 16 | 2.37019993 | 2.96672664 | 3.72325057 | 2.9667 | 0.1148 | -0.052 | 1.9479 |
| male | 16.5 | 2.33776362 | 2.90612324 | 3.62167864 | 2.9061 | 0.1113 | -0.052 | 1.9479 |
| male | 17 | 2.30899887 | 2.8487941 | 3.5229519 | 2.8488 | 0.1074 | -0.052 | 1.9479 |
| male | 17.5 | 2.2825758 | 2.79337774 | 3.42582728 | 2.7934 | 0.1032 | -0.052 | 1.9479 |
| male | 18 | 2.25828896 | 2.73990881 | 3.33077853 | 2.7399 | 0.0988 | -0.052 | 1.9479 |

**Supplementary Table 4:** Percentile table of the derivative left-arm-left-foot (LALF) for Rz/H in ohm/cm

| sex | age | perc_02_5 | perc_50_0 | perc_97_5 | mu | sigma | nu | tau |
| --- | --- | --- | --- | --- | --- | --- | --- | --- |
| female | 5 | 5.74151511 | 6.96938147 | 8.319585 | 6.9694 | 0.0916 | 0.4868 | 1.5894 |
| female | 5.5 | 5.47995656 | 6.69270164 | 8.03009437 | 6.6927 | 0.0944 | 0.4868 | 1.5894 |
| female | 6 | 5.23262454 | 6.42847728 | 7.75087437 | 6.4285 | 0.097 | 0.4868 | 1.5894 |
| female | 6.5 | 5.00479137 | 6.1834278 | 7.49021458 | 6.1834 | 0.0995 | 0.4868 | 1.5894 |
| female | 7 | 4.80142643 | 5.96406133 | 7.25631407 | 5.9641 | 0.1019 | 0.4868 | 1.5894 |
| female | 7.5 | 4.62204573 | 5.77021212 | 7.04935641 | 5.7702 | 0.1042 | 0.4868 | 1.5894 |
| female | 8 | 4.45965292 | 5.59352489 | 6.85946692 | 5.5935 | 0.1063 | 0.4868 | 1.5894 |
| female | 8.5 | 4.30690698 | 5.42504141 | 6.67586744 | 5.425 | 0.1081 | 0.4868 | 1.5894 |
| female | 9 | 4.15682592 | 5.25610894 | 6.4880146 | 5.2561 | 0.1098 | 0.4868 | 1.5894 |
| female | 9.5 | 4.00282439 | 5.0784707 | 6.28575336 | 5.0785 | 0.1113 | 0.4868 | 1.5894 |
| female | 10 | 3.84063831 | 4.88676111 | 6.06245782 | 4.8868 | 0.1126 | 0.4868 | 1.5894 |
| female | 10.5 | 3.67232622 | 4.68368491 | 5.82154405 | 4.6837 | 0.1136 | 0.4868 | 1.5894 |
| female | 11 | 3.50872772 | 4.48326116 | 5.5806101 | 4.4833 | 0.1145 | 0.4868 | 1.5894 |
| female | 11.5 | 3.3633125 | 4.30301108 | 5.36175941 | 4.303 | 0.115 | 0.4868 | 1.5894 |
| female | 12 | 3.24448617 | 4.15403964 | 5.179168 | 4.154 | 0.1153 | 0.4868 | 1.5894 |
| female | 12.5 | 3.15413797 | 4.03906827 | 5.0365241 | 4.0391 | 0.1154 | 0.4868 | 1.5894 |
| female | 13 | 3.09053584 | 3.95609081 | 4.9315353 | 3.9561 | 0.1153 | 0.4868 | 1.5894 |
| female | 13.5 | 3.04877801 | 3.89895584 | 4.85665808 | 3.899 | 0.1148 | 0.4868 | 1.5894 |
| female | 14 | 3.02130904 | 3.8580671 | 4.80001015 | 3.8581 | 0.1142 | 0.4868 | 1.5894 |
| female | 14.5 | 3.00102013 | 3.82440328 | 4.75043197 | 3.8244 | 0.1133 | 0.4868 | 1.5894 |
| female | 15 | 2.98594312 | 3.79552918 | 4.70498205 | 3.7955 | 0.1122 | 0.4868 | 1.5894 |
| female | 15.5 | 2.97884832 | 3.77503259 | 4.66818694 | 3.775 | 0.1108 | 0.4868 | 1.5894 |
| female | 16 | 2.9837522 | 3.7680204 | 4.6463935 | 3.768 | 0.1093 | 0.4868 | 1.5894 |
| female | 16.5 | 3.00120305 | 3.77514215 | 4.6403766 | 3.7751 | 0.1075 | 0.4868 | 1.5894 |
| female | 17 | 3.02715718 | 3.79125855 | 4.64378439 | 3.7913 | 0.1056 | 0.4868 | 1.5894 |
| female | 17.5 | 3.05557702 | 3.80881636 | 4.64739239 | 3.8088 | 0.1035 | 0.4868 | 1.5894 |
| female | 18 | 3.0824516 | 3.82291715 | 4.64534389 | 3.8229 | 0.1013 | 0.4868 | 1.5894 |
| male | 5 | 5.60287795 | 6.51186092 | 7.57762973 | 6.5119 | 0.0769 | -0.054 | 1.9701 |
| male | 5.5 | 5.36225365 | 6.29284986 | 7.39525359 | 6.2928 | 0.0818 | -0.054 | 1.9701 |
| male | 6 | 5.13674612 | 6.08633345 | 7.22277759 | 6.0863 | 0.0868 | -0.054 | 1.9701 |
| male | 6.5 | 4.922911 | 5.88813927 | 7.05493606 | 5.8881 | 0.0916 | -0.054 | 1.9701 |
| male | 7 | 4.71913678 | 5.69620628 | 6.88886388 | 5.6962 | 0.0963 | -0.054 | 1.9701 |
| male | 7.5 | 4.52776574 | 5.51327644 | 6.72751467 | 5.5133 | 0.1008 | -0.054 | 1.9701 |
| male | 8 | 4.35281456 | 5.34430604 | 6.57674322 | 5.3443 | 0.1051 | -0.054 | 1.9701 |
| male | 8.5 | 4.19558605 | 5.19110313 | 6.43875529 | 5.1911 | 0.109 | -0.054 | 1.9701 |
| male | 9 | 4.05297218 | 5.05006089 | 6.3091018 | 5.0501 | 0.1127 | -0.054 | 1.9701 |
| male | 9.5 | 3.91763089 | 4.91218449 | 6.17647484 | 4.9122 | 0.1159 | -0.054 | 1.9701 |
| male | 10 | 3.78150596 | 4.76740156 | 6.02799829 | 4.7674 | 0.1187 | -0.054 | 1.9701 |
| male | 10.5 | 3.64164679 | 4.61198321 | 5.85872152 | 4.612 | 0.1211 | -0.054 | 1.9701 |
| male | 11 | 3.4975787 | 4.44540997 | 5.66789918 | 4.4454 | 0.1229 | -0.054 | 1.9701 |
| male | 11.5 | 3.34754922 | 4.26566623 | 5.45308617 | 4.2657 | 0.1242 | -0.054 | 1.9701 |
| male | 12 | 3.18928844 | 4.07020157 | 5.21136342 | 4.0702 | 0.125 | -0.054 | 1.9701 |
| male | 12.5 | 3.02465625 | 3.86187891 | 4.94697818 | 3.8619 | 0.1253 | -0.054 | 1.9701 |
| male | 13 | 2.86282073 | 3.65303268 | 4.67653944 | 3.653 | 0.1249 | -0.054 | 1.9701 |
| male | 13.5 | 2.71646054 | 3.46051068 | 4.42251477 | 3.4605 | 0.1241 | -0.054 | 1.9701 |
| male | 14 | 2.59635023 | 3.29859245 | 4.20392829 | 3.2986 | 0.1227 | -0.054 | 1.9701 |
| male | 14.5 | 2.50606882 | 3.1721576 | 4.02750263 | 3.1722 | 0.1208 | -0.054 | 1.9701 |
| male | 15 | 2.44024953 | 3.07455536 | 3.88506235 | 3.0746 | 0.1184 | -0.054 | 1.9701 |
| male | 15.5 | 2.39046235 | 2.99525266 | 3.76350439 | 2.9953 | 0.1156 | -0.054 | 1.9701 |
| male | 16 | 2.35040526 | 2.92648692 | 3.65334466 | 2.9265 | 0.1123 | -0.054 | 1.9701 |
| male | 16.5 | 2.31729369 | 2.86497843 | 3.55082478 | 2.865 | 0.1087 | -0.054 | 1.9701 |
| male | 17 | 2.28883459 | 2.80810654 | 3.45305839 | 2.8081 | 0.1047 | -0.054 | 1.9701 |
| male | 17.5 | 2.26350376 | 2.75424236 | 3.35842439 | 2.7542 | 0.1005 | -0.054 | 1.9701 |
| male | 18 | 2.24060384 | 2.70279027 | 3.26657541 | 2.7028 | 0.096 | -0.054 | 1.9701 |

**Supplementary Table 5:** Percentile table of the derivative right-arm-left-foot (RALF) for Rz/H in ohm/cm

| sex | age | perc_02_5 | perc_50_0 | perc_97_5 | mu | sigma | nu | tau |
| --- | --- | --- | --- | --- | --- | --- | --- | --- |
| female | 5 | 5.86608426 | 6.9262736 | 8.1405721 | 6.9263 | 0.0822 | 0.1712 | 1.7571 |
| female | 5.5 | 5.62396006 | 6.68267755 | 7.90149974 | 6.6827 | 0.0853 | 0.1712 | 1.7571 |
| female | 6 | 5.39241348 | 6.44724202 | 7.66763044 | 6.4472 | 0.0883 | 0.1712 | 1.7571 |
| female | 6.5 | 5.17734001 | 6.22712431 | 7.44750598 | 6.2271 | 0.0912 | 0.1712 | 1.7571 |
| female | 7 | 4.98549124 | 6.03070898 | 7.25135848 | 6.0307 | 0.094 | 0.1712 | 1.7571 |
| female | 7.5 | 4.81694027 | 5.85845224 | 7.08006139 | 5.8585 | 0.0966 | 0.1712 | 1.7571 |
| female | 8 | 4.6630092 | 5.70011158 | 6.92150335 | 5.7001 | 0.099 | 0.1712 | 1.7571 |
| female | 8.5 | 4.51624391 | 5.54673479 | 6.76491446 | 5.5467 | 0.1013 | 0.1712 | 1.7571 |
| female | 9 | 4.37020903 | 5.3904717 | 6.60070515 | 5.3905 | 0.1034 | 0.1712 | 1.7571 |
| female | 9.5 | 4.21645866 | 5.2208816 | 6.4159932 | 5.2209 | 0.1053 | 0.1712 | 1.7571 |
| female | 10 | 4.04558572 | 5.02622992 | 6.19619346 | 5.0262 | 0.1069 | 0.1712 | 1.7571 |
| female | 10.5 | 3.85723294 | 4.80599624 | 5.9405184 | 4.806 | 0.1083 | 0.1712 | 1.7571 |
| female | 11 | 3.66724786 | 4.58000552 | 5.67351429 | 4.58 | 0.1094 | 0.1712 | 1.7571 |
| female | 11.5 | 3.49765379 | 4.37606813 | 5.42994281 | 4.3761 | 0.1103 | 0.1712 | 1.7571 |
| female | 12 | 3.36289698 | 4.21268959 | 5.23323335 | 4.2127 | 0.1109 | 0.1712 | 1.7571 |
| female | 12.5 | 3.26665485 | 4.0948765 | 5.0900472 | 4.0949 | 0.1112 | 0.1712 | 1.7571 |
| female | 13 | 3.2052721 | 4.01832637 | 4.99534887 | 4.0183 | 0.1113 | 0.1712 | 1.7571 |
| female | 13.5 | 3.17093658 | 3.97339683 | 4.93732474 | 3.9734 | 0.111 | 0.1712 | 1.7571 |
| female | 14 | 3.15411162 | 3.94820145 | 4.9012839 | 3.9482 | 0.1105 | 0.1712 | 1.7571 |
| female | 14.5 | 3.14432313 | 3.92966588 | 4.87105089 | 3.9297 | 0.1098 | 0.1712 | 1.7571 |
| female | 15 | 3.13618625 | 3.91112321 | 4.83845274 | 3.9111 | 0.1087 | 0.1712 | 1.7571 |
| female | 15.5 | 3.13075993 | 3.89398405 | 4.80536218 | 3.894 | 0.1074 | 0.1712 | 1.7571 |
| female | 16 | 3.13280449 | 3.88424285 | 4.77928975 | 3.8842 | 0.1059 | 0.1712 | 1.7571 |
| female | 16.5 | 3.1451138 | 3.88538499 | 4.76458084 | 3.8854 | 0.1042 | 0.1712 | 1.7571 |
| female | 17 | 3.16486814 | 3.8939225 | 4.75698825 | 3.8939 | 0.1022 | 0.1712 | 1.7571 |
| female | 17.5 | 3.18568918 | 3.90205265 | 4.74706851 | 3.9021 | 0.1 | 0.1712 | 1.7571 |
| female | 18 | 3.20282827 | 3.9041021 | 4.72812065 | 3.9041 | 0.0977 | 0.1712 | 1.7571 |
| male | 5 | 5.72540551 | 6.64723656 | 7.69705511 | 6.6472 | 0.0746 | 0.1211 | 1.8256 |
| male | 5.5 | 5.48603435 | 6.43262313 | 7.51986789 | 6.4326 | 0.0795 | 0.1211 | 1.8256 |
| male | 6 | 5.25931748 | 6.2278961 | 7.34988528 | 6.2279 | 0.0844 | 0.1211 | 1.8256 |
| male | 6.5 | 5.04064774 | 6.02738465 | 7.180025 | 6.0274 | 0.0892 | 0.1211 | 1.8256 |
| male | 7 | 4.82989135 | 5.83065322 | 7.00928252 | 5.8307 | 0.0939 | 0.1211 | 1.8256 |
| male | 7.5 | 4.63191248 | 5.6434064 | 6.84412909 | 5.6434 | 0.0984 | 0.1211 | 1.8256 |
| male | 8 | 4.45235789 | 5.47258512 | 6.6928423 | 5.4726 | 0.1027 | 0.1211 | 1.8256 |
| male | 8.5 | 4.29351064 | 5.32124803 | 6.55923808 | 5.3212 | 0.1068 | 0.1211 | 1.8256 |
| male | 9 | 4.15156391 | 5.18496586 | 6.43797654 | 5.185 | 0.1106 | 0.1211 | 1.8256 |
| male | 9.5 | 4.01652476 | 5.0514042 | 6.31370213 | 5.0514 | 0.114 | 0.1211 | 1.8256 |
| male | 10 | 3.87786403 | 4.90725188 | 6.16949324 | 4.9073 | 0.117 | 0.1211 | 1.8256 |
| male | 10.5 | 3.73302311 | 4.74911883 | 6.00071874 | 4.7491 | 0.1196 | 0.1211 | 1.8256 |
| male | 11 | 3.58327359 | 4.57858862 | 5.80917062 | 4.5786 | 0.1218 | 0.1211 | 1.8256 |
| male | 11.5 | 3.42818035 | 4.39523993 | 5.59433441 | 4.3952 | 0.1234 | 0.1211 | 1.8256 |
| male | 12 | 3.26559305 | 4.19659541 | 5.35328386 | 4.1966 | 0.1246 | 0.1211 | 1.8256 |
| male | 12.5 | 3.09658541 | 3.98446467 | 5.08877077 | 3.9845 | 0.1252 | 0.1211 | 1.8256 |
| male | 13 | 2.92990108 | 3.77068985 | 4.81659275 | 3.7707 | 0.1253 | 0.1211 | 1.8256 |
| male | 13.5 | 2.7783993 | 3.57250058 | 4.55957093 | 3.5725 | 0.1248 | 0.1211 | 1.8256 |
| male | 14 | 2.65348576 | 3.40517439 | 4.33796033 | 3.4052 | 0.1239 | 0.1211 | 1.8256 |
| male | 14.5 | 2.55942067 | 3.27459052 | 4.15978669 | 3.2746 | 0.1224 | 0.1211 | 1.8256 |
| male | 15 | 2.49128886 | 3.17466972 | 4.01763443 | 3.1747 | 0.1204 | 0.1211 | 1.8256 |
| male | 15.5 | 2.44051738 | 3.09461102 | 3.89804967 | 3.0946 | 0.118 | 0.1211 | 1.8256 |
| male | 16 | 2.40034937 | 3.02599343 | 3.79067474 | 3.026 | 0.1152 | 0.1211 | 1.8256 |
| male | 16.5 | 2.36790388 | 2.96537736 | 3.69150679 | 2.9654 | 0.1119 | 0.1211 | 1.8256 |
| male | 17 | 2.34011884 | 2.90914948 | 3.59638556 | 2.9091 | 0.1083 | 0.1211 | 1.8256 |
| male | 17.5 | 2.31485272 | 2.8549142 | 3.50273384 | 2.8549 | 0.1044 | 0.1211 | 1.8256 |
| male | 18 | 2.29154622 | 2.80227372 | 3.41046636 | 2.8023 | 0.1002 | 0.1211 | 1.8256 |

**Supplementary Table 6:** Percentile table of the derivative left-arm-right-foot (LARF) for Rz/H in ohm/cm

| sex | age | perc_02_5 | perc_50_0 | perc_97_5 | mu | sigma | nu | tau |
| --- | --- | --- | --- | --- | --- | --- | --- | --- |
| female | 5 | 0.50421121 | 0.61403039 | 0.77352288 | 0.614 | 0.1082 | -0.7426 | 1.9884 |
| female | 5.5 | 0.48460577 | 0.59029172 | 0.74385527 | 0.5903 | 0.1083 | -0.7426 | 1.9884 |
| female | 6 | 0.46607782 | 0.5678229 | 0.71571421 | 0.5678 | 0.1084 | -0.7426 | 1.9884 |
| female | 6.5 | 0.44913849 | 0.54724748 | 0.68988694 | 0.5472 | 0.1085 | -0.7426 | 1.9884 |
| female | 7 | 0.43444764 | 0.52937072 | 0.66739074 | 0.5294 | 0.1085 | -0.7426 | 1.9884 |
| female | 7.5 | 0.42225083 | 0.51449297 | 0.64860621 | 0.5145 | 0.1085 | -0.7426 | 1.9884 |
| female | 8 | 0.41203688 | 0.50199199 | 0.63274992 | 0.502 | 0.1084 | -0.7426 | 1.9884 |
| female | 8.5 | 0.40297856 | 0.49086013 | 0.61855221 | 0.4909 | 0.1083 | -0.7426 | 1.9884 |
| female | 9 | 0.39389057 | 0.47965415 | 0.60419554 | 0.4797 | 0.1081 | -0.7426 | 1.9884 |
| female | 9.5 | 0.38372287 | 0.46709741 | 0.58807548 | 0.4671 | 0.1079 | -0.7426 | 1.9884 |
| female | 10 | 0.37209356 | 0.45272899 | 0.56961896 | 0.4527 | 0.1077 | -0.7426 | 1.9884 |
| female | 10.5 | 0.35888543 | 0.43641211 | 0.54866431 | 0.4364 | 0.1073 | -0.7426 | 1.9884 |
| female | 11 | 0.34491728 | 0.41914922 | 0.52648392 | 0.4191 | 0.1069 | -0.7426 | 1.9884 |
| female | 11.5 | 0.33191955 | 0.40304823 | 0.50573458 | 0.403 | 0.1065 | -0.7426 | 1.9884 |
| female | 12 | 0.32164531 | 0.39023812 | 0.48908857 | 0.3902 | 0.106 | -0.7426 | 1.9884 |
| female | 12.5 | 0.31477253 | 0.38153621 | 0.47756175 | 0.3815 | 0.1054 | -0.7426 | 1.9884 |
| female | 13 | 0.31142076 | 0.37707853 | 0.4713098 | 0.3771 | 0.1048 | -0.7426 | 1.9884 |
| female | 13.5 | 0.31117102 | 0.37634701 | 0.46966755 | 0.3763 | 0.1041 | -0.7426 | 1.9884 |
| female | 14 | 0.31293025 | 0.37800969 | 0.47095661 | 0.378 | 0.1034 | -0.7426 | 1.9884 |
| female | 14.5 | 0.31520569 | 0.38025763 | 0.47291447 | 0.3803 | 0.1026 | -0.7426 | 1.9884 |
| female | 15 | 0.31683523 | 0.38168934 | 0.47379975 | 0.3817 | 0.1018 | -0.7426 | 1.9884 |
| female | 15.5 | 0.31757075 | 0.38201098 | 0.47325718 | 0.382 | 0.101 | -0.7426 | 1.9884 |
| female | 16 | 0.3181859 | 0.38215835 | 0.47245556 | 0.3822 | 0.1001 | -0.7426 | 1.9884 |
| female | 16.5 | 0.31981266 | 0.38349157 | 0.47307815 | 0.3835 | 0.0991 | -0.7426 | 1.9884 |
| female | 17 | 0.32277067 | 0.38638942 | 0.47558561 | 0.3864 | 0.0981 | -0.7426 | 1.9884 |
| female | 17.5 | 0.3265129 | 0.39019213 | 0.47915861 | 0.3902 | 0.0971 | -0.7426 | 1.9884 |
| female | 18 | 0.33024697 | 0.39395162 | 0.48263192 | 0.394 | 0.0961 | -0.7426 | 1.9884 |
| male | 5 | 0.47246111 | 0.58679555 | 0.72459628 | 0.5868 | 0.1062 | 0.1265 | 1.6219 |
| male | 5.5 | 0.45683472 | 0.57070563 | 0.7086286 | 0.5707 | 0.109 | 0.1265 | 1.6219 |
| male | 6 | 0.4420096 | 0.55521812 | 0.69297731 | 0.5552 | 0.1116 | 0.1265 | 1.6219 |
| male | 6.5 | 0.42778261 | 0.5400838 | 0.6773285 | 0.5401 | 0.1141 | 0.1265 | 1.6219 |
| male | 7 | 0.41437668 | 0.52559123 | 0.66204304 | 0.5256 | 0.1163 | 0.1265 | 1.6219 |
| male | 7.5 | 0.40239026 | 0.51251464 | 0.64810534 | 0.5125 | 0.1183 | 0.1265 | 1.6219 |
| male | 8 | 0.39230945 | 0.50149745 | 0.63634997 | 0.5015 | 0.1201 | 0.1265 | 1.6219 |
| male | 8.5 | 0.38376041 | 0.49208707 | 0.62622581 | 0.4921 | 0.1216 | 0.1265 | 1.6219 |
| male | 9 | 0.3756333 | 0.48287618 | 0.61595491 | 0.4829 | 0.1228 | 0.1265 | 1.6219 |
| male | 9.5 | 0.3668383 | 0.47246958 | 0.60375969 | 0.4725 | 0.1237 | 0.1265 | 1.6219 |
| male | 10 | 0.35671527 | 0.46002102 | 0.58855989 | 0.46 | 0.1243 | 0.1265 | 1.6219 |
| male | 10.5 | 0.34519075 | 0.44544639 | 0.57025814 | 0.4454 | 0.1246 | 0.1265 | 1.6219 |
| male | 11 | 0.33275045 | 0.42939345 | 0.54970784 | 0.4294 | 0.1246 | 0.1265 | 1.6219 |
| male | 11.5 | 0.31987726 | 0.41251533 | 0.52778102 | 0.4125 | 0.1243 | 0.1265 | 1.6219 |
| male | 12 | 0.30681809 | 0.39516548 | 0.50497305 | 0.3952 | 0.1237 | 0.1265 | 1.6219 |
| male | 12.5 | 0.293917 | 0.3778243 | 0.48194437 | 0.3778 | 0.1228 | 0.1265 | 1.6219 |
| male | 13 | 0.28216081 | 0.36179439 | 0.46039986 | 0.3618 | 0.1215 | 0.1265 | 1.6219 |
| male | 13.5 | 0.27280711 | 0.34870883 | 0.44244521 | 0.3487 | 0.12 | 0.1265 | 1.6219 |
| male | 14 | 0.26663546 | 0.33956358 | 0.42934663 | 0.3396 | 0.1183 | 0.1265 | 1.6219 |
| male | 14.5 | 0.26357662 | 0.33425157 | 0.4209498 | 0.3343 | 0.1162 | 0.1265 | 1.6219 |
| male | 15 | 0.26303827 | 0.33199521 | 0.4162478 | 0.332 | 0.1139 | 0.1265 | 1.6219 |
| male | 15.5 | 0.26427703 | 0.33183226 | 0.41400996 | 0.3318 | 0.1114 | 0.1265 | 1.6219 |
| male | 16 | 0.26667735 | 0.33297372 | 0.4132374 | 0.333 | 0.1087 | 0.1265 | 1.6219 |
| male | 16.5 | 0.26991653 | 0.33500838 | 0.41341466 | 0.335 | 0.1058 | 0.1265 | 1.6219 |
| male | 17 | 0.27374675 | 0.33762558 | 0.41415944 | 0.3376 | 0.1028 | 0.1265 | 1.6219 |
| male | 17.5 | 0.27805046 | 0.34068151 | 0.41530118 | 0.3407 | 0.0996 | 0.1265 | 1.6219 |
| male | 18 | 0.2827844 | 0.34412648 | 0.41678742 | 0.3441 | 0.0963 | 0.1265 | 1.6219 |

**Supplementary Table 7:** Percentile table of the derivative right-arm-right-foot (RARF) for Xc/H in ohm/cm

| sex | age | perc_02_5 | perc_50_0 | perc_97_5 | mu | sigma | nu | tau |
| --- | --- | --- | --- | --- | --- | --- | --- | --- |
| female | 5 | 0.45011651 | 0.57822288 | 0.75877646 | 0.5782 | 0.1322 | -0.3127 | 1.923 |
| female | 5.5 | 0.43645693 | 0.5586696 | 0.7300339 | 0.5587 | 0.1303 | -0.3127 | 1.923 |
| female | 6 | 0.42314025 | 0.53978339 | 0.70254319 | 0.5398 | 0.1284 | -0.3127 | 1.923 |
| female | 6.5 | 0.41039336 | 0.5218306 | 0.67660834 | 0.5218 | 0.1266 | -0.3127 | 1.923 |
| female | 7 | 0.3986351 | 0.50531958 | 0.65284549 | 0.5053 | 0.125 | -0.3127 | 1.923 |
| female | 7.5 | 0.3881929 | 0.4906392 | 0.63171219 | 0.4906 | 0.1233 | -0.3127 | 1.923 |
| female | 8 | 0.37901386 | 0.47769679 | 0.61304595 | 0.4777 | 0.1218 | -0.3127 | 1.923 |
| female | 8.5 | 0.37061581 | 0.46586334 | 0.59600399 | 0.4659 | 0.1204 | -0.3127 | 1.923 |
| female | 9 | 0.36212701 | 0.45402978 | 0.5791445 | 0.454 | 0.119 | -0.3127 | 1.923 |
| female | 9.5 | 0.35276904 | 0.441216 | 0.56120841 | 0.4412 | 0.1176 | -0.3127 | 1.923 |
| female | 10 | 0.34227015 | 0.42708388 | 0.54176572 | 0.4271 | 0.1164 | -0.3127 | 1.923 |
| female | 10.5 | 0.33051911 | 0.41149813 | 0.52064769 | 0.4115 | 0.1151 | -0.3127 | 1.923 |
| female | 11 | 0.31808827 | 0.39517364 | 0.49876009 | 0.3952 | 0.114 | -0.3127 | 1.923 |
| female | 11.5 | 0.30631304 | 0.37976519 | 0.47818334 | 0.3798 | 0.1129 | -0.3127 | 1.923 |
| female | 12 | 0.29666139 | 0.36707863 | 0.46116908 | 0.3671 | 0.1118 | -0.3127 | 1.923 |
| female | 12.5 | 0.28969983 | 0.35779378 | 0.44853937 | 0.3578 | 0.1108 | -0.3127 | 1.923 |
| female | 13 | 0.2854755 | 0.35194694 | 0.44030704 | 0.3519 | 0.1098 | -0.3127 | 1.923 |
| female | 13.5 | 0.28364919 | 0.34910061 | 0.43589605 | 0.3491 | 0.1089 | -0.3127 | 1.923 |
| female | 14 | 0.2834439 | 0.34828402 | 0.43407287 | 0.3483 | 0.108 | -0.3127 | 1.923 |
| female | 14.5 | 0.28372244 | 0.34809212 | 0.43307471 | 0.3481 | 0.1072 | -0.3127 | 1.923 |
| female | 15 | 0.28373474 | 0.34760345 | 0.43175282 | 0.3476 | 0.1064 | -0.3127 | 1.923 |
| female | 15.5 | 0.28348642 | 0.34682656 | 0.43011968 | 0.3468 | 0.1057 | -0.3127 | 1.923 |
| female | 16 | 0.2836808 | 0.34662251 | 0.42924345 | 0.3466 | 0.105 | -0.3127 | 1.923 |
| female | 16.5 | 0.28516309 | 0.34802144 | 0.43039571 | 0.348 | 0.1043 | -0.3127 | 1.923 |
| female | 17 | 0.28808612 | 0.35120542 | 0.43379488 | 0.3512 | 0.1037 | -0.3127 | 1.923 |
| female | 17.5 | 0.29193228 | 0.35554033 | 0.43865301 | 0.3555 | 0.1032 | -0.3127 | 1.923 |
| female | 18 | 0.29599875 | 0.36017022 | 0.44391371 | 0.3602 | 0.1027 | -0.3127 | 1.923 |
| male | 5 | 0.45211306 | 0.57504701 | 0.74095798 | 0.575 | 0.1225 | -0.2127 | 1.6198 |
| male | 5.5 | 0.43848517 | 0.5593873 | 0.72318498 | 0.5594 | 0.1241 | -0.2127 | 1.6198 |
| male | 6 | 0.4253057 | 0.54402105 | 0.70540738 | 0.544 | 0.1255 | -0.2127 | 1.6198 |
| male | 6.5 | 0.41254902 | 0.52892284 | 0.68759602 | 0.5289 | 0.1267 | -0.2127 | 1.6198 |
| male | 7 | 0.40037328 | 0.51430469 | 0.67003399 | 0.5143 | 0.1277 | -0.2127 | 1.6198 |
| male | 7.5 | 0.38924752 | 0.50078139 | 0.65353507 | 0.5008 | 0.1285 | -0.2127 | 1.6198 |
| male | 8 | 0.37972407 | 0.4890786 | 0.63906439 | 0.4891 | 0.1291 | -0.2127 | 1.6198 |
| male | 8.5 | 0.37163587 | 0.47899599 | 0.626379 | 0.479 | 0.1295 | -0.2127 | 1.6198 |
| male | 9 | 0.36402942 | 0.46931614 | 0.61390197 | 0.4693 | 0.1296 | -0.2127 | 1.6198 |
| male | 9.5 | 0.35585155 | 0.45869128 | 0.59988438 | 0.4587 | 0.1295 | -0.2127 | 1.6198 |
| male | 10 | 0.34649745 | 0.44635503 | 0.5833436 | 0.4464 | 0.1292 | -0.2127 | 1.6198 |
| male | 10.5 | 0.33600517 | 0.43237538 | 0.56439759 | 0.4324 | 0.1286 | -0.2127 | 1.6198 |
| male | 11 | 0.32466931 | 0.41715534 | 0.54360951 | 0.4172 | 0.1278 | -0.2127 | 1.6198 |
| male | 11.5 | 0.31263931 | 0.40091408 | 0.52130724 | 0.4009 | 0.1268 | -0.2127 | 1.6198 |
| male | 12 | 0.29998558 | 0.38377161 | 0.49769318 | 0.3838 | 0.1256 | -0.2127 | 1.6198 |
| male | 12.5 | 0.28704727 | 0.3661926 | 0.47341788 | 0.3662 | 0.1241 | -0.2127 | 1.6198 |
| male | 13 | 0.27469324 | 0.34931195 | 0.44998993 | 0.3493 | 0.1224 | -0.2127 | 1.6198 |
| male | 13.5 | 0.26396743 | 0.33447117 | 0.42916067 | 0.3345 | 0.1206 | -0.2127 | 1.6198 |
| male | 14 | 0.25560039 | 0.32259328 | 0.41211359 | 0.3226 | 0.1185 | -0.2127 | 1.6198 |
| male | 14.5 | 0.24984028 | 0.31397449 | 0.39920589 | 0.314 | 0.1163 | -0.2127 | 1.6198 |
| male | 15 | 0.24654626 | 0.30841257 | 0.39014779 | 0.3084 | 0.1139 | -0.2127 | 1.6198 |
| male | 15.5 | 0.24534755 | 0.30541645 | 0.38428319 | 0.3054 | 0.1113 | -0.2127 | 1.6198 |
| male | 16 | 0.2457602 | 0.30435959 | 0.38079362 | 0.3044 | 0.1086 | -0.2127 | 1.6198 |
| male | 16.5 | 0.247275 | 0.30459369 | 0.3788471 | 0.3046 | 0.1059 | -0.2127 | 1.6198 |
| male | 17 | 0.2493384 | 0.30542796 | 0.37757574 | 0.3054 | 0.103 | -0.2127 | 1.6198 |
| male | 17.5 | 0.25163682 | 0.30647834 | 0.37650813 | 0.3065 | 0.1 | -0.2127 | 1.6198 |
| male | 18 | 0.25413116 | 0.3077017 | 0.37560032 | 0.3077 | 0.0969 | -0.2127 | 1.6198 |

**Supplementary Table 8:** Percentile table of the derivative right-arm-left-arm (RALA) for Xc/H in ohm/cm

| sex | age | perc_02_5 | perc_50_0 | perc_97_5 | mu | sigma | nu | tau |
| --- | --- | --- | --- | --- | --- | --- | --- | --- |
| female | 5 | 0.44296221 | 0.56069682 | 0.73100465 | 0.5607 | 0.1264 | -0.472 | 1.9018 |
| female | 5.5 | 0.42725178 | 0.54112664 | 0.70601241 | 0.5411 | 0.1267 | -0.472 | 1.9018 |
| female | 6 | 0.41214657 | 0.52230309 | 0.68196242 | 0.5223 | 0.127 | -0.472 | 1.9018 |
| female | 6.5 | 0.3977567 | 0.50436292 | 0.65902851 | 0.5044 | 0.1274 | -0.472 | 1.9018 |
| female | 7 | 0.38429923 | 0.48757975 | 0.63756537 | 0.4876 | 0.1277 | -0.472 | 1.9018 |
| female | 7.5 | 0.37185768 | 0.472059 | 0.61770982 | 0.4721 | 0.128 | -0.472 | 1.9018 |
| female | 8 | 0.36022913 | 0.45754309 | 0.59912467 | 0.4575 | 0.1283 | -0.472 | 1.9018 |
| female | 8.5 | 0.34923418 | 0.44380428 | 0.58151174 | 0.4438 | 0.1286 | -0.472 | 1.9018 |
| female | 9 | 0.33863843 | 0.43054488 | 0.56448107 | 0.4305 | 0.1289 | -0.472 | 1.9018 |
| female | 9.5 | 0.32816088 | 0.41740773 | 0.54756444 | 0.4174 | 0.1291 | -0.472 | 1.9018 |
| female | 10 | 0.31758942 | 0.40412317 | 0.53040814 | 0.4041 | 0.1294 | -0.472 | 1.9018 |
| female | 10.5 | 0.30692902 | 0.39069788 | 0.51302137 | 0.3907 | 0.1296 | -0.472 | 1.9018 |
| female | 11 | 0.29669491 | 0.3777887 | 0.49626816 | 0.3778 | 0.1297 | -0.472 | 1.9018 |
| female | 11.5 | 0.28778016 | 0.36653482 | 0.48164822 | 0.3665 | 0.1299 | -0.472 | 1.9018 |
| female | 12 | 0.2810188 | 0.35800136 | 0.47056588 | 0.358 | 0.13 | -0.472 | 1.9018 |
| female | 12.5 | 0.27664203 | 0.35248588 | 0.46341729 | 0.3525 | 0.1301 | -0.472 | 1.9018 |
| female | 13 | 0.27442427 | 0.34970348 | 0.45983202 | 0.3497 | 0.1302 | -0.472 | 1.9018 |
| female | 13.5 | 0.27380122 | 0.34893688 | 0.4588699 | 0.3489 | 0.1302 | -0.472 | 1.9018 |
| female | 14 | 0.27399212 | 0.34919229 | 0.45922612 | 0.3492 | 0.1302 | -0.472 | 1.9018 |
| female | 14.5 | 0.27432879 | 0.34961916 | 0.45978379 | 0.3496 | 0.1302 | -0.472 | 1.9018 |
| female | 15 | 0.27447047 | 0.34978432 | 0.45997517 | 0.3498 | 0.1302 | -0.472 | 1.9018 |
| female | 15.5 | 0.27454777 | 0.34985567 | 0.46002341 | 0.3499 | 0.1302 | -0.472 | 1.9018 |
| female | 16 | 0.27508016 | 0.35049672 | 0.46080364 | 0.3505 | 0.1301 | -0.472 | 1.9018 |
| female | 16.5 | 0.27650831 | 0.35227054 | 0.46305877 | 0.3523 | 0.13 | -0.472 | 1.9018 |
| female | 17 | 0.27888106 | 0.3552409 | 0.46687528 | 0.3552 | 0.13 | -0.472 | 1.9018 |
| female | 17.5 | 0.28189913 | 0.35902835 | 0.47175745 | 0.359 | 0.1299 | -0.472 | 1.9018 |
| female | 18 | 0.28515562 | 0.36311689 | 0.47703098 | 0.3631 | 0.1298 | -0.472 | 1.9018 |
| male | 5 | 0.43363376 | 0.5430236 | 0.67449277 | 0.543 | 0.1121 | 0.167 | 1.9279 |
| male | 5.5 | 0.41686446 | 0.52562468 | 0.65705883 | 0.5256 | 0.1155 | 0.167 | 1.9279 |
| male | 6 | 0.40105002 | 0.50904489 | 0.64024762 | 0.509 | 0.1187 | 0.167 | 1.9279 |
| male | 6.5 | 0.38628359 | 0.49341539 | 0.62422959 | 0.4934 | 0.1218 | 0.167 | 1.9279 |
| male | 7 | 0.37272009 | 0.47895134 | 0.60928785 | 0.479 | 0.1247 | 0.167 | 1.9279 |
| male | 7.5 | 0.36048551 | 0.4658364 | 0.5956725 | 0.4658 | 0.1274 | 0.167 | 1.9279 |
| male | 8 | 0.34962076 | 0.4541476 | 0.58350194 | 0.4541 | 0.13 | 0.167 | 1.9279 |
| male | 8.5 | 0.33991006 | 0.44362759 | 0.57246469 | 0.4436 | 0.1323 | 0.167 | 1.9279 |
| male | 9 | 0.33067492 | 0.43340671 | 0.56145004 | 0.4334 | 0.1343 | 0.167 | 1.9279 |
| male | 9.5 | 0.32115025 | 0.42248729 | 0.54916518 | 0.4225 | 0.1361 | 0.167 | 1.9279 |
| male | 10 | 0.31076902 | 0.41012199 | 0.53463124 | 0.4101 | 0.1376 | 0.167 | 1.9279 |
| male | 10.5 | 0.29939898 | 0.39613585 | 0.51761473 | 0.3961 | 0.1389 | 0.167 | 1.9279 |
| male | 11 | 0.2874468 | 0.38107458 | 0.49883381 | 0.3811 | 0.1399 | 0.167 | 1.9279 |
| male | 11.5 | 0.27542633 | 0.36563743 | 0.47922229 | 0.3656 | 0.1405 | 0.167 | 1.9279 |
| male | 12 | 0.26364905 | 0.35026292 | 0.45938281 | 0.3503 | 0.1409 | 0.167 | 1.9279 |
| male | 12.5 | 0.25235348 | 0.33529606 | 0.43980046 | 0.3353 | 0.1409 | 0.167 | 1.9279 |
| male | 13 | 0.24238732 | 0.32189141 | 0.42202327 | 0.3219 | 0.1407 | 0.167 | 1.9279 |
| male | 13.5 | 0.23493712 | 0.31164748 | 0.40817477 | 0.3116 | 0.1401 | 0.167 | 1.9279 |
| male | 14 | 0.23078542 | 0.3056119 | 0.3996397 | 0.3056 | 0.1393 | 0.167 | 1.9279 |
| male | 14.5 | 0.22972674 | 0.30350664 | 0.39604911 | 0.3035 | 0.1382 | 0.167 | 1.9279 |
| male | 15 | 0.23088289 | 0.30415675 | 0.39585449 | 0.3042 | 0.1368 | 0.167 | 1.9279 |
| male | 15.5 | 0.23326911 | 0.30625109 | 0.39733568 | 0.3063 | 0.1351 | 0.167 | 1.9279 |
| male | 16 | 0.23619395 | 0.30887482 | 0.39930062 | 0.3089 | 0.1332 | 0.167 | 1.9279 |
| male | 16.5 | 0.23933846 | 0.31161095 | 0.40121397 | 0.3116 | 0.1311 | 0.167 | 1.9279 |
| male | 17 | 0.24265598 | 0.31440293 | 0.40301178 | 0.3144 | 0.1287 | 0.167 | 1.9279 |
| male | 17.5 | 0.24626871 | 0.31741388 | 0.40491279 | 0.3174 | 0.1262 | 0.167 | 1.9279 |
| male | 18 | 0.2502662 | 0.32076226 | 0.40707577 | 0.3208 | 0.1234 | 0.167 | 1.9279 |

**Supplementary Table 9:** Percentile table of the derivative right-foot-left-foot (RFLF) for Xc/H in ohm/cm

| sex | age | perc_02_5 | perc_50_0 | perc_97_5 | mu | sigma | nu | tau |
| --- | --- | --- | --- | --- | --- | --- | --- | --- |
| female | 5 | 0.49754388 | 0.62816074 | 0.83635239 | 0.6282 | 0.1305 | -0.7936 | 1.9778 |
| female | 5.5 | 0.48391005 | 0.60798195 | 0.80358763 | 0.608 | 0.1275 | -0.7936 | 1.9778 |
| female | 6 | 0.47026017 | 0.58816044 | 0.77216054 | 0.5882 | 0.1247 | -0.7936 | 1.9778 |
| female | 6.5 | 0.45665966 | 0.56874449 | 0.74203301 | 0.5687 | 0.1222 | -0.7936 | 1.9778 |
| female | 7 | 0.44359249 | 0.55030368 | 0.71385466 | 0.5503 | 0.1198 | -0.7936 | 1.9778 |
| female | 7.5 | 0.4315507 | 0.53341192 | 0.68827629 | 0.5334 | 0.1176 | -0.7936 | 1.9778 |
| female | 8 | 0.42056621 | 0.51807174 | 0.66521206 | 0.5181 | 0.1155 | -0.7936 | 1.9778 |
| female | 8.5 | 0.41035897 | 0.50390631 | 0.64410287 | 0.5039 | 0.1136 | -0.7936 | 1.9778 |
| female | 9 | 0.40025329 | 0.49006221 | 0.62380104 | 0.4901 | 0.1119 | -0.7936 | 1.9778 |
| female | 9.5 | 0.38944476 | 0.47554074 | 0.6029992 | 0.4755 | 0.1103 | -0.7936 | 1.9778 |
| female | 10 | 0.37739269 | 0.45967575 | 0.58083266 | 0.4597 | 0.1088 | -0.7936 | 1.9778 |
| female | 10.5 | 0.36387233 | 0.44219132 | 0.55694082 | 0.4422 | 0.1074 | -0.7936 | 1.9778 |
| female | 11 | 0.34967498 | 0.42404533 | 0.5325166 | 0.424 | 0.1062 | -0.7936 | 1.9778 |
| female | 11.5 | 0.33653543 | 0.4073293 | 0.5101592 | 0.4073 | 0.105 | -0.7936 | 1.9778 |
| female | 12 | 0.32622193 | 0.39416067 | 0.49247607 | 0.3942 | 0.104 | -0.7936 | 1.9778 |
| female | 12.5 | 0.31929495 | 0.38518877 | 0.4802263 | 0.3852 | 0.1031 | -0.7936 | 1.9778 |
| female | 13 | 0.31555394 | 0.38014666 | 0.47303095 | 0.3801 | 0.1022 | -0.7936 | 1.9778 |
| female | 13.5 | 0.31435375 | 0.37823838 | 0.46986509 | 0.3782 | 0.1015 | -0.7936 | 1.9778 |
| female | 14 | 0.31475795 | 0.37832553 | 0.46929269 | 0.3783 | 0.1009 | -0.7936 | 1.9778 |
| female | 14.5 | 0.31569482 | 0.37911469 | 0.46969886 | 0.3791 | 0.1004 | -0.7936 | 1.9778 |
| female | 15 | 0.31631388 | 0.37958391 | 0.46981527 | 0.3796 | 0.1 | -0.7936 | 1.9778 |
| female | 15.5 | 0.3163725 | 0.37944359 | 0.4692851 | 0.3794 | 0.0996 | -0.7936 | 1.9778 |
| female | 16 | 0.31634783 | 0.37926748 | 0.46881968 | 0.3793 | 0.0994 | -0.7936 | 1.9778 |
| female | 16.5 | 0.31694949 | 0.37990714 | 0.46947253 | 0.3799 | 0.0993 | -0.7936 | 1.9778 |
| female | 17 | 0.31823619 | 0.38143416 | 0.47133379 | 0.3814 | 0.0993 | -0.7936 | 1.9778 |
| female | 17.5 | 0.31972063 | 0.38326679 | 0.4736885 | 0.3833 | 0.0993 | -0.7936 | 1.9778 |
| female | 18 | 0.32091244 | 0.38482019 | 0.47581909 | 0.3848 | 0.0995 | -0.7936 | 1.9778 |
| male | 5 | 0.50422655 | 0.61590029 | 0.75729135 | 0.6159 | 0.101 | -0.1597 | 1.6293 |
| male | 5.5 | 0.48763912 | 0.59983268 | 0.7430839 | 0.5998 | 0.1046 | -0.1597 | 1.6293 |
| male | 6 | 0.47147476 | 0.58383003 | 0.72844293 | 0.5838 | 0.108 | -0.1597 | 1.6293 |
| male | 6.5 | 0.45558884 | 0.5677043 | 0.71310129 | 0.5677 | 0.1113 | -0.1597 | 1.6293 |
| male | 7 | 0.44019527 | 0.55171688 | 0.69735957 | 0.5517 | 0.1143 | -0.1597 | 1.6293 |
| male | 7.5 | 0.42580759 | 0.53651736 | 0.68202649 | 0.5365 | 0.117 | -0.1597 | 1.6293 |
| male | 8 | 0.41294672 | 0.52278132 | 0.66796783 | 0.5228 | 0.1194 | -0.1597 | 1.6293 |
| male | 8.5 | 0.40157856 | 0.51049172 | 0.65518014 | 0.5105 | 0.1216 | -0.1597 | 1.6293 |
| male | 9 | 0.39095581 | 0.49872017 | 0.64248627 | 0.4987 | 0.1234 | -0.1597 | 1.6293 |
| male | 9.5 | 0.38015207 | 0.48629694 | 0.62838281 | 0.4863 | 0.1248 | -0.1597 | 1.6293 |
| male | 10 | 0.36850026 | 0.47237657 | 0.6117776 | 0.4724 | 0.1259 | -0.1597 | 1.6293 |
| male | 10.5 | 0.3558579 | 0.45678891 | 0.59245985 | 0.4568 | 0.1266 | -0.1597 | 1.6293 |
| male | 11 | 0.34250971 | 0.43992222 | 0.57096012 | 0.4399 | 0.1269 | -0.1597 | 1.6293 |
| male | 11.5 | 0.32869015 | 0.42211069 | 0.54775645 | 0.4221 | 0.1268 | -0.1597 | 1.6293 |
| male | 12 | 0.31448518 | 0.40350533 | 0.52310242 | 0.4035 | 0.1264 | -0.1597 | 1.6293 |
| male | 12.5 | 0.3002452 | 0.3846014 | 0.49770699 | 0.3846 | 0.1255 | -0.1597 | 1.6293 |
| male | 13 | 0.28705322 | 0.36683088 | 0.47349041 | 0.3668 | 0.1243 | -0.1597 | 1.6293 |
| male | 13.5 | 0.27622996 | 0.35191335 | 0.45272233 | 0.3519 | 0.1227 | -0.1597 | 1.6293 |
| male | 14 | 0.26863236 | 0.3409498 | 0.43683841 | 0.3409 | 0.1207 | -0.1597 | 1.6293 |
| male | 14.5 | 0.26431118 | 0.33399192 | 0.42589428 | 0.334 | 0.1185 | -0.1597 | 1.6293 |
| male | 15 | 0.26270071 | 0.33030046 | 0.41892132 | 0.3303 | 0.1159 | -0.1597 | 1.6293 |
| male | 15.5 | 0.26299854 | 0.32884263 | 0.41458532 | 0.3288 | 0.113 | -0.1597 | 1.6293 |
| male | 16 | 0.26448179 | 0.32869927 | 0.41171543 | 0.3287 | 0.1099 | -0.1597 | 1.6293 |
| male | 16.5 | 0.2666844 | 0.32928623 | 0.40958232 | 0.3293 | 0.1066 | -0.1597 | 1.6293 |
| male | 17 | 0.26929348 | 0.33022062 | 0.40772303 | 0.3302 | 0.103 | -0.1597 | 1.6293 |
| male | 17.5 | 0.27224217 | 0.33142922 | 0.40606697 | 0.3314 | 0.0993 | -0.1597 | 1.6293 |
| male | 18 | 0.27557567 | 0.33297653 | 0.40471288 | 0.333 | 0.0955 | -0.1597 | 1.6293 |

**Supplementary Table 10:** Percentile table of the derivative left-arm-left-foot (LALF) for Xc/H in ohm/cm

| sex | age | perc_02_5 | perc_50_0 | perc_97_5 | mu | sigma | nu | tau |
| --- | --- | --- | --- | --- | --- | --- | --- | --- |
| female | 5 | 0.50204955 | 0.62607682 | 0.81746625 | 0.6261 | 0.1236 | -0.7782 | 2.0912 |
| female | 5.5 | 0.48598973 | 0.60431963 | 0.78577765 | 0.6043 | 0.1219 | -0.7782 | 2.0912 |
| female | 6 | 0.47019817 | 0.58311414 | 0.7552571 | 0.5831 | 0.1202 | -0.7782 | 2.0912 |
| female | 6.5 | 0.45480382 | 0.56259954 | 0.72603513 | 0.5626 | 0.1187 | -0.7782 | 2.0912 |
| female | 7 | 0.44033492 | 0.54340821 | 0.6988797 | 0.5434 | 0.1173 | -0.7782 | 2.0912 |
| female | 7.5 | 0.42726499 | 0.52610132 | 0.67446211 | 0.5261 | 0.1159 | -0.7782 | 2.0912 |
| female | 8 | 0.41552347 | 0.51056888 | 0.6525927 | 0.5106 | 0.1147 | -0.7782 | 2.0912 |
| female | 8.5 | 0.40468065 | 0.49626357 | 0.63253164 | 0.4963 | 0.1135 | -0.7782 | 2.0912 |
| female | 9 | 0.39399807 | 0.48226631 | 0.61307866 | 0.4823 | 0.1123 | -0.7782 | 2.0912 |
| female | 9.5 | 0.38273111 | 0.46765834 | 0.59304895 | 0.4677 | 0.1113 | -0.7782 | 2.0912 |
| female | 10 | 0.37049497 | 0.45196668 | 0.57183447 | 0.452 | 0.1103 | -0.7782 | 2.0912 |
| female | 10.5 | 0.35725168 | 0.4351438 | 0.54937016 | 0.4351 | 0.1094 | -0.7782 | 2.0912 |
| female | 11 | 0.34374814 | 0.41809695 | 0.52679444 | 0.4181 | 0.1085 | -0.7782 | 2.0912 |
| female | 11.5 | 0.33140963 | 0.4025523 | 0.50626713 | 0.4026 | 0.1077 | -0.7782 | 2.0912 |
| female | 12 | 0.32162057 | 0.39017821 | 0.48986133 | 0.3902 | 0.107 | -0.7782 | 2.0912 |
| female | 12.5 | 0.31481682 | 0.38148708 | 0.47819032 | 0.3815 | 0.1063 | -0.7782 | 2.0912 |
| female | 13 | 0.31088737 | 0.37632921 | 0.47103884 | 0.3763 | 0.1057 | -0.7782 | 2.0912 |
| female | 13.5 | 0.30929405 | 0.37404112 | 0.46755465 | 0.374 | 0.1051 | -0.7782 | 2.0912 |
| female | 14 | 0.30911817 | 0.373504 | 0.46632493 | 0.3735 | 0.1046 | -0.7782 | 2.0912 |
| female | 14.5 | 0.30942658 | 0.3735868 | 0.46593079 | 0.3736 | 0.1041 | -0.7782 | 2.0912 |
| female | 15 | 0.30963399 | 0.37358243 | 0.46548902 | 0.3736 | 0.1037 | -0.7782 | 2.0912 |
| female | 15.5 | 0.30970283 | 0.37344647 | 0.46494527 | 0.3734 | 0.1034 | -0.7782 | 2.0912 |
| female | 16 | 0.31011548 | 0.37376136 | 0.46502559 | 0.3738 | 0.1031 | -0.7782 | 2.0912 |
| female | 16.5 | 0.31148947 | 0.37527158 | 0.46665624 | 0.3753 | 0.1029 | -0.7782 | 2.0912 |
| female | 17 | 0.31395318 | 0.37813215 | 0.4700302 | 0.3781 | 0.1027 | -0.7782 | 2.0912 |
| female | 17.5 | 0.31711443 | 0.38187204 | 0.47456401 | 0.3819 | 0.1026 | -0.7782 | 2.0912 |
| female | 18 | 0.32041366 | 0.38581995 | 0.47942761 | 0.3858 | 0.1026 | -0.7782 | 2.0912 |
| male | 5 | 0.49233993 | 0.60675444 | 0.75201721 | 0.6068 | 0.1053 | -0.1266 | 1.6391 |
| male | 5.5 | 0.47741188 | 0.5917588 | 0.73790947 | 0.5918 | 0.1082 | -0.1266 | 1.6391 |
| male | 6 | 0.4629814 | 0.57699707 | 0.72364576 | 0.577 | 0.111 | -0.1266 | 1.6391 |
| male | 6.5 | 0.44881432 | 0.56217491 | 0.70883932 | 0.5622 | 0.1136 | -0.1266 | 1.6391 |
| male | 7 | 0.43491352 | 0.54729364 | 0.69347484 | 0.5473 | 0.116 | -0.1266 | 1.6391 |
| male | 7.5 | 0.42156797 | 0.53271983 | 0.67800731 | 0.5327 | 0.1181 | -0.1266 | 1.6391 |
| male | 8 | 0.40920472 | 0.51900424 | 0.66314032 | 0.519 | 0.12 | -0.1266 | 1.6391 |
| male | 8.5 | 0.39790498 | 0.50626713 | 0.6490398 | 0.5063 | 0.1216 | -0.1266 | 1.6391 |
| male | 9 | 0.38712744 | 0.49383633 | 0.63485701 | 0.4938 | 0.123 | -0.1266 | 1.6391 |
| male | 9.5 | 0.3761548 | 0.48080899 | 0.61944008 | 0.4808 | 0.124 | -0.1266 | 1.6391 |
| male | 10 | 0.36441692 | 0.46646738 | 0.60187334 | 0.4665 | 0.1247 | -0.1266 | 1.6391 |
| male | 10.5 | 0.35173568 | 0.45059855 | 0.58189855 | 0.4506 | 0.1251 | -0.1266 | 1.6391 |
| male | 11 | 0.33836298 | 0.43354785 | 0.55999053 | 0.4335 | 0.1252 | -0.1266 | 1.6391 |
| male | 11.5 | 0.32454725 | 0.41566362 | 0.53663998 | 0.4157 | 0.125 | -0.1266 | 1.6391 |
| male | 12 | 0.31041491 | 0.39714298 | 0.51215094 | 0.3971 | 0.1245 | -0.1266 | 1.6391 |
| male | 12.5 | 0.29632642 | 0.37848534 | 0.48722233 | 0.3785 | 0.1236 | -0.1266 | 1.6391 |
| male | 13 | 0.28334274 | 0.36108168 | 0.46369723 | 0.3611 | 0.1224 | -0.1266 | 1.6391 |
| male | 13.5 | 0.27274825 | 0.34659219 | 0.44374365 | 0.3466 | 0.121 | -0.1266 | 1.6391 |
| male | 14 | 0.26533136 | 0.33602239 | 0.4286583 | 0.336 | 0.1192 | -0.1266 | 1.6391 |
| male | 14.5 | 0.26103524 | 0.32928629 | 0.41831716 | 0.3293 | 0.1172 | -0.1266 | 1.6391 |
| male | 15 | 0.25923971 | 0.32557972 | 0.41167432 | 0.3256 | 0.115 | -0.1266 | 1.6391 |
| male | 15.5 | 0.2591725 | 0.32391307 | 0.40745756 | 0.3239 | 0.1125 | -0.1266 | 1.6391 |
| male | 16 | 0.26021107 | 0.32349585 | 0.40466186 | 0.3235 | 0.1098 | -0.1266 | 1.6391 |
| male | 16.5 | 0.26201729 | 0.32390253 | 0.40275398 | 0.3239 | 0.1069 | -0.1266 | 1.6391 |
| male | 17 | 0.26436376 | 0.32485149 | 0.40138904 | 0.3249 | 0.1038 | -0.1266 | 1.6391 |
| male | 17.5 | 0.26715909 | 0.32623326 | 0.40044104 | 0.3262 | 0.1006 | -0.1266 | 1.6391 |
| male | 18 | 0.27035909 | 0.32799803 | 0.39985943 | 0.328 | 0.0973 | -0.1266 | 1.6391 |

**Supplementary Table 11:** Percentile table of the derivative right-arm-left-foot (RALF) for Xc/H in ohm/cm

| sex | age | perc_02_5 | perc_50_0 | perc_97_5 | mu | sigma | nu | tau |
| --- | --- | --- | --- | --- | --- | --- | --- | --- |
| female | 5 | 0.44058823 | 0.55204213 | 0.71373494 | 0.552 | 0.1229 | -0.5408 | 2.0725 |
| female | 5.5 | 0.42592537 | 0.53234636 | 0.68605848 | 0.5323 | 0.1215 | -0.5408 | 2.0725 |
| female | 6 | 0.41199203 | 0.5137287 | 0.66006713 | 0.5137 | 0.1201 | -0.5408 | 2.0725 |
| female | 6.5 | 0.39914038 | 0.49660727 | 0.63625821 | 0.4966 | 0.1189 | -0.5408 | 2.0725 |
| female | 7 | 0.3877483 | 0.48143056 | 0.61516646 | 0.4814 | 0.1177 | -0.5408 | 2.0725 |
| female | 7.5 | 0.37792359 | 0.46831094 | 0.59689674 | 0.4683 | 0.1165 | -0.5408 | 2.0725 |
| female | 8 | 0.36933019 | 0.45681302 | 0.58086035 | 0.4568 | 0.1155 | -0.5408 | 2.0725 |
| female | 8.5 | 0.36153601 | 0.44638787 | 0.56633344 | 0.4464 | 0.1145 | -0.5408 | 2.0725 |
| female | 9 | 0.35395543 | 0.43630253 | 0.55236806 | 0.4363 | 0.1135 | -0.5408 | 2.0725 |
| female | 9.5 | 0.34595539 | 0.42577132 | 0.53795928 | 0.4258 | 0.1126 | -0.5408 | 2.0725 |
| female | 10 | 0.33702344 | 0.41416213 | 0.52230467 | 0.4142 | 0.1118 | -0.5408 | 2.0725 |
| female | 10.5 | 0.32672854 | 0.40094653 | 0.5047385 | 0.4009 | 0.111 | -0.5408 | 2.0725 |
| female | 11 | 0.31544957 | 0.38659136 | 0.48585032 | 0.3866 | 0.1102 | -0.5408 | 2.0725 |
| female | 11.5 | 0.30450968 | 0.3727166 | 0.4676724 | 0.3727 | 0.1095 | -0.5408 | 2.0725 |
| female | 12 | 0.29554379 | 0.36131553 | 0.45269252 | 0.3613 | 0.1088 | -0.5408 | 2.0725 |
| female | 12.5 | 0.2893991 | 0.35341116 | 0.44217149 | 0.3534 | 0.1082 | -0.5408 | 2.0725 |
| female | 13 | 0.28622676 | 0.34917442 | 0.43630098 | 0.3492 | 0.1076 | -0.5408 | 2.0725 |
| female | 13.5 | 0.28557997 | 0.34804849 | 0.43436624 | 0.348 | 0.107 | -0.5408 | 2.0725 |
| female | 14 | 0.28650717 | 0.34886563 | 0.43489693 | 0.3489 | 0.1065 | -0.5408 | 2.0725 |
| female | 14.5 | 0.28787077 | 0.35023714 | 0.43615601 | 0.3502 | 0.106 | -0.5408 | 2.0725 |
| female | 15 | 0.28878759 | 0.3510888 | 0.43680587 | 0.3511 | 0.1056 | -0.5408 | 2.0725 |
| female | 15.5 | 0.28913094 | 0.3512688 | 0.43666076 | 0.3513 | 0.1052 | -0.5408 | 2.0725 |
| female | 16 | 0.28964775 | 0.35168637 | 0.43685344 | 0.3517 | 0.1049 | -0.5408 | 2.0725 |
| female | 16.5 | 0.2912598 | 0.35346092 | 0.43877447 | 0.3535 | 0.1046 | -0.5408 | 2.0725 |
| female | 17 | 0.2942465 | 0.35693106 | 0.44284314 | 0.3569 | 0.1043 | -0.5408 | 2.0725 |
| female | 17.5 | 0.29821152 | 0.36161636 | 0.44846376 | 0.3616 | 0.1042 | -0.5408 | 2.0725 |
| female | 18 | 0.30249732 | 0.36672142 | 0.4546527 | 0.3667 | 0.104 | -0.5408 | 2.0725 |
| male | 5 | 0.43773667 | 0.53437113 | 0.65027445 | 0.5344 | 0.0996 | 0.0809 | 1.7971 |
| male | 5.5 | 0.42325714 | 0.52034589 | 0.63753726 | 0.5203 | 0.1031 | 0.0809 | 1.7971 |
| male | 6 | 0.40951385 | 0.50683426 | 0.62501806 | 0.5068 | 0.1064 | 0.0809 | 1.7971 |
| male | 6.5 | 0.3964628 | 0.49378258 | 0.61263944 | 0.4938 | 0.1095 | 0.0809 | 1.7971 |
| male | 7 | 0.38422891 | 0.48135007 | 0.60059117 | 0.4814 | 0.1124 | 0.0809 | 1.7971 |
| male | 7.5 | 0.37311005 | 0.46991979 | 0.58935131 | 0.4699 | 0.115 | 0.0809 | 1.7971 |
| male | 8 | 0.36342166 | 0.45990623 | 0.57944938 | 0.4599 | 0.1174 | 0.0809 | 1.7971 |
| male | 8.5 | 0.35506791 | 0.45120838 | 0.57077256 | 0.4512 | 0.1194 | 0.0809 | 1.7971 |
| male | 9 | 0.34727381 | 0.44285586 | 0.56210193 | 0.4429 | 0.1211 | 0.0809 | 1.7971 |
| male | 9.5 | 0.3391068 | 0.43366384 | 0.55193141 | 0.4337 | 0.1225 | 0.0809 | 1.7971 |
| male | 10 | 0.3299334 | 0.42282265 | 0.5392248 | 0.4228 | 0.1236 | 0.0809 | 1.7971 |
| male | 10.5 | 0.31966772 | 0.41022782 | 0.52385109 | 0.4102 | 0.1243 | 0.0809 | 1.7971 |
| male | 11 | 0.30872066 | 0.3964231 | 0.50652184 | 0.3964 | 0.1246 | 0.0809 | 1.7971 |
| male | 11.5 | 0.29758074 | 0.38206256 | 0.48810425 | 0.3821 | 0.1245 | 0.0809 | 1.7971 |
| male | 12 | 0.28657778 | 0.36760238 | 0.4692216 | 0.3676 | 0.124 | 0.0809 | 1.7971 |
| male | 12.5 | 0.27599256 | 0.35343764 | 0.45042262 | 0.3534 | 0.1232 | 0.0809 | 1.7971 |
| male | 13 | 0.26658179 | 0.3405698 | 0.43302601 | 0.3406 | 0.122 | 0.0809 | 1.7971 |
| male | 13.5 | 0.25935979 | 0.33031592 | 0.41873588 | 0.3303 | 0.1205 | 0.0809 | 1.7971 |
| male | 14 | 0.25498577 | 0.32351797 | 0.4086267 | 0.3235 | 0.1186 | 0.0809 | 1.7971 |
| male | 14.5 | 0.25338415 | 0.32006496 | 0.40254465 | 0.3201 | 0.1164 | 0.0809 | 1.7971 |
| male | 15 | 0.25399482 | 0.31922661 | 0.399549 | 0.3192 | 0.114 | 0.0809 | 1.7971 |
| male | 15.5 | 0.25609538 | 0.32007498 | 0.39845997 | 0.3201 | 0.1112 | 0.0809 | 1.7971 |
| male | 16 | 0.25906187 | 0.32181936 | 0.39828633 | 0.3218 | 0.1082 | 0.0809 | 1.7971 |
| male | 16.5 | 0.26255262 | 0.32403411 | 0.3985063 | 0.324 | 0.105 | 0.0809 | 1.7971 |
| male | 17 | 0.26640753 | 0.32652669 | 0.39889544 | 0.3265 | 0.1016 | 0.0809 | 1.7971 |
| male | 17.5 | 0.27063615 | 0.32931664 | 0.3994929 | 0.3293 | 0.098 | 0.0809 | 1.7971 |
| male | 18 | 0.27527897 | 0.33246077 | 0.400382 | 0.3325 | 0.0942 | 0.0809 | 1.7971 |

**Supplementary Table 12:** Percentile table of the derivative left-arm-right-foot (LARF) for Xc/H in ohm/cm

| comparison | sex | mean_diff_  bias | mean_diff_  lower.limit | mean_diff_  upper.limit | pvalue | CI_lower_  lower.limit | CI_lower_  upper.limit | CI_upper_  lower.limit | CI_upper_  upper.limit | CI_lower_  mean.diffs | CI_upper_  mean.diffs |
| --- | --- | --- | --- | --- | --- | --- | --- | --- | --- | --- | --- |
| Rz/H-SDS RARF vs LALF | female | -0.02 | -0.71 | 0.67 | 0.063 | -0.74 | 0.64 | -0.68 | 0.71 | -0.04 | 0.00 |
| Rz/H-SDS RARF vs LALF | male | 0.00 | -0.60 | 0.60 | 0.800 | -0.62 | 0.57 | -0.57 | 0.63 | -0.01 | 0.02 |
| Xc/H-SDS RARF vs LALF | female | 0.03 | -0.88 | 0.94 | 0.030 | -0.93 | 0.90 | -0.84 | 0.98 | 0.00 | 0.05 |
| Xc/H-SDS RARF vs LALF | male | 0.02 | -0.81 | 0.86 | 0.033 | -0.85 | 0.82 | -0.78 | 0.90 | 0.00 | 0.05 |
| Rz/H-SDS RALF vs LARF | female | 0.02 | -0.83 | 0.87 | 0.095 | -0.87 | 0.83 | -0.79 | 0.91 | 0.00 | 0.04 |
| Rz/H-SDS RALF vs LARF | male | 0.00 | -0.68 | 0.68 | 0.943 | -0.71 | 0.65 | -0.65 | 0.71 | -0.02 | 0.02 |
| Xc/H-SDS RALF vs LARF | female | -0.05 | -1.12 | 1.02 | <0.001 | -1.17 | 0.96 | -1.07 | 1.07 | -0.08 | -0.03 |
| Xc/H-SDS RALF vs LARF | male | -0.01 | -0.92 | 0.89 | 0.330 | -0.96 | 0.85 | -0.88 | 0.93 | -0.04 | 0.01 |
| BIACORPUS vs Gätjens FFM (kg) | female | 0.11 | -3.57 | 3.78 | 0.034 | -3.74 | 3.61 | -3.40 | 3.95 | 0.01 | 0.21 |
| BIACORPUS vs Gätjens FFM (kg) | male | 1.50 | -2.62 | 5.62 | <0.001 | -2.80 | 5.43 | -2.44 | 5.80 | 1.39 | 1.61 |
| BIACORPUS vs Gätjens FFM (%) | female | 0.01 | -0.08 | 0.09 | <0.001 | -0.08 | 0.09 | -0.08 | 0.10 | 0.00 | 0.01 |
| BIACORPUS vs Gätjens FFM (%) | male | 0.03 | -0.06 | 0.12 | <0.001 | -0.06 | 0.11 | -0.06 | 0.12 | 0.03 | 0.03 |
| BIACORPUS vs Gätjens FM (kg) | female | -0.13 | -3.81 | 3.56 | 0.011 | -3.98 | 3.38 | -3.64 | 3.73 | -0.23 | -0.03 |
| BIACORPUS vs Gätjens FM (kg) | male | -1.50 | -5.61 | 2.61 | <0.001 | -5.80 | 2.43 | -5.43 | 2.79 | -1.61 | -1.40 |
| BIACORPUS vs Gätjens FM (%) | female | 24.44 | 3.17 | 45.72 | <0.001 | 2.17 | 44.73 | 4.16 | 46.71 | 23.87 | 25.02 |
| BIACORPUS vs Gätjens FM (%) | male | 18.00 | -1.38 | 37.39 | <0.001 | -2.24 | 36.52 | -0.52 | 38.25 | 17.51 | 18.50 |
| BIA vs Slaugher | female | 0.11 | -10.88 | 11.10 | 0.493 | -11.41 | 10.57 | -10.35 | 11.62 | -0.20 | 0.41 |
| BIA vs Slaugher | male | 4.14 | -11.78 | 20.10 | <0.001 | -12.60 | 19.24 | -10.97 | 20.87 | 3.67 | 4.61 |

**Supplementary Table 13:** Statistical measures for all Bland-Altman analyses.

| comparison | sex | age_  group | n | mean_diff_  bias | mean_diff_  lower.limit | mean_diff_  upper.limit | pvalue | CI_lower_  lower.limit | CI_lower_  upper.limit | CI_upper_  lower.limit | | CI_upper_  upper.limit | CI_lower_  mean.diffs | CI_upper_  mean.diffs |
| --- | --- | --- | --- | --- | --- | --- | --- | --- | --- | --- | --- | --- | --- | --- |
| BIA vs Slaugher | female | (-Inf,9] | 168 | -4.39 | -13.16 | 4.39 | <0.001 | -14.37 | 3.18 | -11.96 | | 5.59 | -5.08 | -3.69 |
| BIA vs Slaugher | female | (9,12] | 233 | 2.51 | -9.95 | 14.97 | <0.001 | -11.41 | 13.51 | -8.49 | 16.42 | | 1.66 | 3.35 |
| BIA vs Slaugher | female | (12,16] | 377 | 0.73 | -6.02 | 7.49 | <0.001 | -6.64 | 6.87 | -5.41 | | 8.11 | 0.38 | 1.09 |
| BIA vs Slaugher | female | (16, Inf] | 172 | -0.74 | -7.18 | 5.70 | 0.005 | -8.06 | 4.82 | -6.29 | | 6.59 | -1.25 | -0.23 |
| BIA vs Slaugher | male | (-Inf,9] | 203 | -3.16 | -11.08 | 4.76 | <0.001 | -12.07 | 3.76 | -10.08 | | 5.75 | -3.74 | -2.58 |
| BIA vs Slaugher | male | (9,12] | 273 | 3.65 | -9.27 | 16.57 | <0.001 | -10.72 | 15.11 | -7.81 | | 18.02 | 2.81 | 4.491 |
| BIA vs Slaugher | male | (12,16] | 397 | 3.82 | -3.40 | 11.03 | <0.001 | -4.25 | 10.17 | -2.53 | | 11.88 | 3.32 | 4.31 |
| BIA vs Slaugher | male | (16, Inf] | 155 | -0.43 | -9.96 | 9.09 | 0.449 | -11.92 | 7.13 | -7.99 | | 11.06 | -1.57 | 0.70 |
| BIACORPUS vs Gätjens | female | (-Inf,9] | 240 | 23.56 | 3.61 | 43.51 | <0.001 | 1.33 | 41.23 | 5.89 | | 45.79 | 22.24 | 24.87 |
| BIACORPUS vs Gätjens | female | (9,12] | 347 | 20.41 | -3.86 | 44.68 | <0.001 | -6.15 | 42.39 | -1.56 | | 46.98 | 19.09 | 21.74 |
| BIACORPUS vs Gätjens | female | (12,16] | 593 | 25.40 | 5.419 | 45.38 | <0.001 | 3.98 | 43.94 | 6.86 | | 46.82 | 24.57 | 26.23 |
| BIACORPUS vs Gätjens | female | (16, Inf] | 243 | 28.76 | 12.21 | 45.32 | <0.001 | 10.32 | 43.44 | 14.09 | | 47.20 | 27.68 | 29.85 |
| BIACORPUS vs Gätjens | male | (-Inf,9] | 265 | 19.67 | 1.14 | 38.20 | <0.001 | -0.87 | 36.19 | 3.14 | | 40.21 | 18.51 | 20.83 |
| BIACORPUS vs Gätjens | male | (9,12] | 418 | 18.03 | -4.61 | 40.66 | <0.001 | -6.55 | 38.72 | -2.67 | | 42.61 | 16.91 | 19.15 |
| BIACORPUS vs Gätjens | male | (12,16] | 640 | 17.26 | -1.47 | 35.98 | <0.001 | -2.76 | 34.69 | -0.18 | | 37.27 | 16.51 | 18.00 |
| BIACORPUS vs Gätjens | male | (16, Inf] | 213 | 18.18 | 3.73 | 32.63 | <0.001 | 1.99 | 30.89 | 5.47 | | 34.37 | 17.17 | 19.19 |

**Supplementary Table 14:** Age-stratified analyses of FM estimation methods.
